# Oleoylethanolamide facilitates PPARα and TFEB signaling and attenuates Aβ pathology in a mouse model of Alzheimer’s disease

**DOI:** 10.1186/s13024-023-00648-x

**Published:** 2023-08-15

**Authors:** Michele M. Comerota, Manasee Gedam, Wen Xiong, Feng Jin, Lisheng Deng, Meng C. Wang, Jin Wang, Hui Zheng

**Affiliations:** 1 Huffington Center on Aging, Baylor College of Medicine, One Baylor Plaza, Houston, TX 77030 USA; 2 Translational Biology and Molecular Medicine Graduate Program, Houston, TX USA; 3 Department of Pharmacology and Chemical Biology, Houston, TX USA; 4 Department of Molecular and Human Genetics, Houston, TX USA; 5 Howard Hughes Medical Institute, Baylor College of Medicine, Houston, TX USA; 6 Present Address: HHMI Janelia Research Campus, Ashburn, VA USA

**Keywords:** Alzheimer’s disease, Microglia, Oleoylethanolamide, PPARα, TFEB

## Abstract

**Background:**

Age is the strongest risk factor for the development of Alzheimer’s disease (AD). Besides the pathological hallmarks of β-amyloid (Aβ) plaques and neurofibrillary tangles, emerging evidence demonstrates a critical role of microglia and neuroinflammation in AD pathogenesis. Oleoylethanolamide (OEA) is an endogenous lipid amide that has been shown to promote lifespan and healthspan in *C. elegans* through regulation of lysosome-to-nucleus signaling and cellular metabolism. The goal of our study was to determine the role of OEA in the mediation of microglial activity and AD pathology using its stable analog, KDS-5104.

**Methods:**

We used primary microglial cultures and genetic and pharmacological approaches to examine the signaling mechanisms and functional roles of OEA in mediating Aβ phagocytosis and clearance, lipid metabolism and inflammasome formation. Further, we tested the effect of OEA in vivo in acute LPS-induced neuroinflammation and by chronic treatment of 5xFAD mice.

**Results:**

We found that OEA activates PPARα signaling and its downstream cell-surface receptor CD36 activity. In addition, OEA promotes TFEB lysosomal function in a PPARα-dependent but mTORC1-independent manner, the combination of which leads to enhanced microglial Aβ uptake and clearance. These are associated with the suppression of LPS-induced lipid droplet accumulation and inflammasome activation. Chronic treatment of 5xFAD mice with KDS-5104 restored dysregulated lipid profiles, reduced reactive gliosis and Aβ pathology and rescued cognitive impairments.

**Conclusion:**

Together, our study provides support that augmenting OEA-mediated lipid signaling may offer therapeutic benefit against aging and AD through modulating lipid metabolism and microglia phagocytosis and clearance.

**Supplementary Information:**

The online version contains supplementary material available at 10.1186/s13024-023-00648-x.

## Background

Alzheimer’s disease (AD) is the most prevalent age-related neurodegenerative disorder characterized by the accumulation of amyloid beta (Aβ) plaques and neurofibrillary tangles [1]. These pathological hallmarks are accompanied by prominent changes of glial cells in the brain. Genome-wide association studies implicate a contributing role of microglia and its associated pathways such as endocytosis and phagocytosis, lipid metabolism and immune response in the etiology of late-onset AD [2]. Besides the genetic evidence, age is known to be the greatest risk factor. The underlying mechanisms for the age influence are likely complex as aging is known to elicit a multitude of changes at cellular, organelle and system levels. Accordingly, compounds that delay aging and promote longevity may prove efficacious in combating AD.

Oleoylethanolamide (OEA) is an evolutionarily conserved endogenous lipid that has been shown to extend the lifespan and healthspan of *C. elegans* through lysosome-to-nucleus signaling and activation of metabolic gene expression [3]. In the mammalian system, OEA is produced in both peripheral tissues and the central nervous system (CNS). Intriguingly, a recent lipidomics analysis identified OEA and other fatty acid ethanolamide as a lipid class downregulated in the cerebral spinal fluid and plasma of AD patients compared to non-demented controls [4], raising the possibility that OEA and related lipids may influence AD progression and their circulating levels may serve as useful biomarkers.

The effect of OEA in feeding regulation has been attributed to its binding to the peroxisome proliferator activated receptor alpha (PPARα) [5, 6], a ligand activated nuclear receptor that, upon dimerization with the retinoid X receptor (RXR), acts as a potent transcription factor to activate downstream targets involved in energy homeostasis, lipid metabolism, autophagy, and inflammation [7]. Besides PPARα, there are two additional PPAR isoforms, PPARβ/δ and PPARγ. They are expressed in both peripheral tissues and the CNS [8–10].Through similar signaling mechanisms, the PPARs play critical roles in cellular metabolism and their dysregulation have been linked to multiple diseases such as type 2 diabetes, cancer and Alzheimer’s disease [11–13]. Synthetic PPARα agonists have been shown to provide beneficial effects in AD mouse models by acting on APP processing and Aβ metabolism [14, 15], autophagy and lysosomal pathway [16–18], lipid peroxidation [19], and neuroinflammation [20, 21]. PPARα may exert its effect through crosstalk with PPARβ/δ and PPARγ [12] or with other transcription factors, among them, the transcription factor EB (TFEB). TFEB is a master regulator of autophagy and lysosome biogenesis that coordinates lysosome nutrient status with mTOR-dependent phosphorylation and nuclear signaling [22]. We and others have reported a potent role of TFEB in mitigating Aβ and tau pathologies through both the autophagy-lysosomal pathway and phagocytosis [23–27]. PPARα and TFEB have an intricate network of regulation in which they share common upstream inducers and downstream targets [7]. Relevant to AD, Raha et al. reported an astrocytic PPARα-TFEB pathway in regulating Aβ clearance [18]. However, a possible role of OEA in the brain in physiological and disease conditions is poorly understood.

OEA is a lipid amide that can be hydrolyzed by fatty-acid amide hydrolase (FAAH) [28]. To increase the stability of OEA, Astarita et al. developed an analog, KDS-5104, that is a functional mimetic of OEA and resistant to enzymatic hydrolysis [28]. Using the analog in the current study, we present evidence that OEA/KDS-5104 activates PPARα downstream target CD36 to enhance phagocytosis and TFEB to promote lysosomal clearance, the latter is mTOR-independent. These concerted activities lead to potent microglial Aβ and lipid uptake and clearance and suppression of LPS-induced inflammasome activation. Administration of KDS-5104 to the 5xFAD mouse model of AD reversed lipid profile alterations, and microglia and astrocyte reactivity in the brain. These changes were accompanied by attenuated Aβ pathology, improved synaptic integrity and cognitive function in the AD model.

## Methods

### Mice and treatment

Mice were housed 3–4 mice per cage in a pathogen free mouse facility with ad libitum access to food and water on a 12 h light/dark cycle. All experiments included approximately equal ratio of male and female mice. The number of mice used for each experiment was specified in the figure legend. All procedures were performed in accordance with NIH guidelines and approval of the Baylor College of Medicine Institutional Animal Care and Use Committee (IACUC).

In vivo LPS treatment was performed as previously described [29]. Briefly, PPARαKO (Jackson Laboratory; Strain #:008154) and WT mice received a pretreatment of KDS-5104 (10 mg/kg, i.p.) or vehicle. 24 h post KDS-5104 treatment, mice received a co-treatment of KDS-5104 (10 mg/kg, i.p.) and LPS (2 mg/kg, i.p.). 18 h after the LPS injection, tissue was collected and fixed or frozen for further analysis.

For efficacy studies, two-month-old 5xFAD and WT littermate were treated with KDS-5104 (10 mg/kg, i.p.) or vehicle for three days a week for a total of 8 weeks. At the end of the treatment, behavioral assays were performed, and mice were sacrificed. The brains were perfused with PBS, dissected, and frozen or fixed for qPCR and biochemical analysis and immunostaining respectively.

### In vitro cultures

The cellular model systems used include primary microglia cultures, BV2 cells and HeLa stable cell line containing the knockout of the three TFEB family genes; *TFEB*, *TFE3* and *MITF* (TKO) [30].

Primary microglia monocultures were prepared from mixed gender PPARαKO and WT pups as previously described [31, 32]. Briefly, cortices were isolated from PPARα and wild type newborn pups (P0-P1) and cut finely in dissection media [Hank’s balanced salt solution (HBSS), 10% mM HEPES, and 1% (v/v) penicillin/streptomycin]. Next, tissue was digested with 2.5% trypsin for 15 min at 37^o^C before the addition of trypsin inhibitor (1 mg/ml) for 1 min. Tissue was then centrifuged for 5 min at 1500 rpm. Next, the pellet was triturated, resuspended in complete media (DMEM with 10% fetal bovine serum and 1% (v/v) penicillin/ streptomycin) and plated onto poly-d-lysine (PDL)–coated T-75 flasks at 50,000 cells/cm^2^ to produce mixed glial cultures. For microglia monocultures, mixed glia cultures were allowed to grow for one week, then microglia were separated from the mixed glia by shaking the confluent flask at 250 RPM for 2.5 h twice, 48 h between the two sessions. Following the final shaking, the flasks were tapped on a table and the floated cells, primarily microglia, were collected and seeded in PDL coated 12-well plate or glass coverslips at a concentration of 50,000 cells/cm^2^. Experiments were performed 24–48 h post seeding.

For KDS-5104 treatment, cells were plated 24 h prior to treatment. KDS-5104 was added to the complete media at a concentration of 10 µM unless otherwise stated. Eight hours after addition of KDS-5104, the cells were fixed or collected for further analysis. For PPARα antagonist experiments, cells were pretreated with 10 µM of GW6471 (Cayman Chemical) for 3 h prior to KDS-5104 treatment.

For LPS treatment, primary microglia or BV2 cell cultures were plated in 24 well plates with PDL-coated glass cover slips. Cells were treated with 10 µM of KDS-5104 or vehicle. Following an 8-hour incubation, media was replaced by LPS (5 µg/ml) and KDS-5104 (10 µM) containing media. Eighteen hours following LPS application, cells were fixed in 4% PFA in preparation for immunostaining procedures. For lipid droplet assay, following fixation cells were stained with BODIPY (Invitrogen) and washed with PBS three times. Coverslips were mounted and imaged using a Leica STELLARIS confocal microscope.

### FACS based isolation of microglia from adult mouse brain

FACS sorting of microglia was performed as previously described with minor modifications [33]. Briefly, 9-month-old mice were perfused with PBS, brains extracted and gently minced with sterile razor blades. The tissue was digested in papain (Worthington Biochemical) and DNase (Worthington Biochemical), then titrated 5–6 times by a sterile fire-polished glass Pasteur pipette. Next, ice-cold HBSS+ (HBSS with 2mM EDTA and 0.5% BSA) was added and the suspension was pelleted at 310 g for 5 min at 4 °C. The pellet was resuspended in 1ml of HBSS+, triturated 5–6 additional times, and centrifuged. After centrifugation, the supernatant was filtered through a 40 μm cell strainer (BD Biosciences) and further centrifuged at 310 g for 5 min at 4 °C. The resulting pellet was resuspended in 20% 4 °C Percoll PLUS (Millipore-Sigma) in 1× PBS and centrifuged at 310 g at 4 °C for 20 min. The resulting pellet was incubated in 500ul HBSS + containing 1:100 Mouse BD Fc Block (BD Biosciences). Then with the following antibodies: rat anti–CD45-BV421 (1:500, BD Biosciences), rat anti–CD11b-FITC (1:500, BD Biosciences). Microglia population was gated and sorted based on CD45^mid^ and CD11b^+^ expression. Sorting was performed using BD Biosciences Aria II on the 100 μm nozzle. Cells were sorted into 1.7 ml Eppendorf tubes coated with 200 µl HBSS+, followed by lysis of pellets in Qiagen RLT buffer containing 1% β-mercaptoethanol for downstream RNA analysis.

### Phagocytosis assay

Phagocytosis assays were performed in primary microglia cultures as previously described [34]. Fluorescent latex beads were preprepared in FBS for 1 h at 37^o^C at a 1:5 ratio. The beads/FBS mixture was then added to prewarmed complete media (1:1000). Beads containing media were added to cells for 1 h, then removed and washed thoroughly with PBS. Cells were then fixed in 4% PFA and prepared for immunostaining. Cells were analyzed for percentage of microglia with beads internalization. For Aβ uptake and degradation, microglia were pretreated with CD36 neutralizing antibody (2 µg/ml; Abcam) as previously described [35]. Following blocking of CD36, microglia were treated with KDS-5104 for 8 h (10 µM). Meanwhile, fluorescently labelled Aβ_42_ (Cayman Chemical) was incubated in complete media for 37^o^C for 1 h at a concentration of 500 nM. Treated microglia were then incubated in Aβ containing media for 1 h at 37^o^C. Media were removed and cells washed. A portion of the cells were fixed at time point 0 and prepared for imaging. The remaining cells were fed with Aβ free fresh complete media for 1, 2 and 4 h. The cells were fixed, stained, and imaged on confocal with a 40x objective and the fluorescence of Aβ per cell was analyzed using the ImageJ software (NIH).

### Western blotting

Brain tissue or cell pellets were lysed and homogenized in RIPA buffer containing protease and phosphatase inhibitors. Homogenates were centrifuged at 10,000 g for 15 min at 4^o^C and supernatant was collected. Bicinchoninic acid analysis (Thermo Fisher Scientific) was used to determine and normalize protein concentrations. Protein separation was performed by electrophoresis using 10–15% SDS-polyacrylamide gels. Following separation, proteins were transferred to a nitrocellulose membrane. Nonspecific binding was blocked by 5% BSA in tris-buffered saline then primary antibodies were incubated overnight at 4^o^C. Primary antibodies were used at the following concentrations; PPARα (1:1000; Cell Signaling), APP (1:1000; recognizes APP-FL and CTF, Cell Signaling), β-actin (1:10,000; Sigma-Aldrich), pS6k (1:1000; Cell Signaling), total S6k (1:1000, Cell Signaling), pAKT (1:1000, Cell Signaling), total AKT (1:1000, Cell Signaling) and γ tubulin (1:1000, Cell Signaling). After primary antibody incubations, secondary antibodies; IR-680–conjugated goat anti-mouse or goat anti-rabbit (1:10,000; Molecular Probes) and IRDye 800–conjugated donkey anti-rabbit or donkey anti-mouse (1:10,000; LI-COR) were used. LI-COR Odyssey machine (LI-COR) was used to image the membranes. The Western blot bands were quantified using the ImageJ software.

### RNA isolation, reverse transcription and qPCR

Total RNA was isolated from cells, human and mouse brain tissues by lysing in Qiagen RLT buffer with 1% β-mercaptoethanol and processed using the RNeasy Mini kit (Qiagen). Reverse transcription was carried out on the isolated RNA using iScript Reverse Transcription Supermix (Bio-Rad). The qPCR analyses were performed using iTaq Universal SYBR Green master mix (Bio-Rad) on a CFX384 Touch Real-Time PCR Detection System. Housekeeping genes 18s and GAPDH were used as controls. Relative levels of gene expression were quantified by the Bio-Rad CFX manager. Heatmaps were constructed using GraphPad Prism.

### Immunofluorescence

Cells were fixed in 4% PFA and prepared for immunocytochemistry. Briefly, blocking buffer (0.2% BSA, 0.5% Triton X-100, and 0.05% Tween 20 in PBS) was used to block nonspecific binding sites for 1 h at room temperature. Next, cells were incubated with primary antibodies: Iba-1 (1:800 or 1:500; Wako or Novus Biologicals, respectively), CD36 (1:1000; Abcam), LAMP1 (1:500; BD Biosciences). Following primary antibody incubation, cells were incubated with appropriate secondary antibodies (Alexa Fluor 488, 555, or 647; Invitrogen). The nucleus was then stained with 4′,6-diamidino-2-phenylindole (DAPI). Glass cover slips were then mounted and imaged under the confocal microscope. For lysotracker staining, prior to fixation, cells were incubated in complete media containing lysotracker dye (50 nM; Thermo Fisher Scientific) for 30 min at 37^o^C. Cells were thoroughly washed in PBS and fixed for 15 min in 4% PFA. Coverslips were mounted and imaged.

For mouse brain tissue, immunohistochemistry was performed on free floating brain sections. Briefly, mice were perfused with saline, brains quickly extracted and fixed in 4% PFA at 4^o^C overnight. Brains were then transferred to 30% sucrose for 48 h and microtome-cut into 30 μm thick sections. Free floating sections were incubated with primary antibodies: 6E10 (1:1000; BioLegend), Iba1 (1:800 or 1:500; Wako or Novus Biologicals, respectively), GFAP (1:1000; Sigma), CD68 (1:500; BioLegend), PSD95 (1:200; Millipore), synaptophysin (1:500; Abcam). Next, appropriate secondary antibodies (Alexa Fluor 488, 594, or 647; Invitrogen) were used followed by incubation with DAPI. Three sections (including hippocampus and cortex) per mouse brain with five to seven mice per group were used. Brain sections from treatment groups were coded prior to imaging to reduce imaging bias.

### Image quantification

#### Synaptic co-localization

Synaptic marker co-localization analysis was performed with the Imaris software (Oxford Instruments) as described previously [29, 32]. Briefly, synaptophysin (Abcam) and PSD95 (Millipore), respective markers for pre- and post-synaptic terminals, were stained in mouse brain sections as described above. Sections were imaged with a 63X oil objective with a 4.0 digital zoom on the Leica STELLARIS confocal microscope. 5 μm thick Z stacks with 0.2 μm step size were obtained. Using the Imaris ‘Spots’ feature, puncta from each channel were analyzed by generation of spot representation (automatic generation with consistent manual adjustment for all images for accuracy). Total number of spots for each channel were recorded. Spots were then analyzed using the ‘Co-localize Spots’ MATLAB plugin, defining co-localization if the center of the pre- and post-synaptic puncta were within 200 nm.

#### Lysosome characterization

For analysis of LAMP1 and lysotracker images (obtained with 63x objective, 2x digital zoom) the Cells feature of Imaris was used. Cell borders and nucleus were defined followed by the generation of representative spots for each marked lysosome (automatic generation with consistent manual adjustment for all images for accuracy). Spots were then analyzed for size, fluorescent intensity, proximity to nucleus and number per cell.

### Lipidomics

Brain tissue from 4-month-old WT-vehicle, WT-KDS-5104, 5xFAD-vehicle and 5xFAD-KDS-5104 mice were weighed and normalized. Tissue was then ground using a bead beater. 10 mg of each sample was collected and homogenized in 50 mM ammonium acetate solution. 10 µl of splash lipidomix Mass Spec Standard (Avanti, 330,707) was added to each sample. Following standard addition, lipids were extracted using methanol, metyl terrt-Butyl Ether (MTBE) and water. The samples were then dried in a vacufuge and resuspended in 110 µl isopropanol and methanol (50:50, vol/vol). The samples were analyzed using a Vanquish UPLC and a Lumos orbitrap mass spectrometer (Thermo Fisher Scientific). Analysis of lipidomic data were performed using Lipidsearch software (Thermo Fisher Scientific). The statistical analysis was done by MetaboAnalyst 5.0.

### Behavioral assays

At the conclusion of animal treatments with vehicle or KDS-5104, mice underwent behavioral testing. Mice treatment groups were coded and blinded to researcher performing and analyzing results. Prior to each assay, mice were habituated to the test room for 30 min.

*Novel object recognition*: The novel object recognition protocol included three phases: habituation, a training, and object recognition. All three phase are performed in a Plexiglass arene (measuring 22 cm by 44 cm). The habitation phase included one 5-minute session, in which the animals were allowed to freely explore the arena. Twenty-four hours post habituation, the animals underwent training, in which the mice were placed in the arena with two identical objects. The animals were allowed to freely explore the objects for 5 min. One day after the training phase, the mice underwent testing, in which the mice were placed in the same arena with one object previously explored in the training phase, the familiar object, and one novel object differing in color and shape but sharing a common size and volume. The animals were allowed to freely explore the objects for 5 min. The time spend exploring each object was measured by the ANY-maze software. Exploration of an object was defined by head orientation directed toward the object or physical contact with the object. The object discrimination ratio (ODR) was calculated by the following formula: ODR = (time exploring specified object) / (time exploring novel object + time exploring familiar object) × 100.

#### Fear conditioning

The fear conditioning protocol involved a training phase, context test, and a cued test. During the training phase, the mice were placed in the conditioning chamber and allowed to freely explore. At 3 min, an auditory stimulus was presented for 30 min (80-dB white noise) followed by the administration of a foot shock (0.8 mA, 2 s). This was repeated a second time at the 5-min mark. Following training, the mice were then returned to their original housing cages for 24 h before performing context and cue testing. For the context test, each mouse was returned to the same chamber (same geometric shape of chamber, lights, scents, and auditory sounds) for 5 min with no stimulation, freezing was recorded. The cue test was then performed, one hour after the context test. For the cue test, mice were placed in an altered chamber consisting of a different geometric shape, flooring, light brightness, and scent compared to the previous chamber used for training. After 3 min, the auditory stimulus was presented for 3 min. The software FreezeFrame3 and FreezeView (San Diego Instruments) was used to record and analyze the percent freezing in each trial. Methods for open field, grip strength and rotarod tests are described in Supplementary Materials.

### Statistical analysis

All data were analyzed with GraphPad Prism version 6 and presented as means ± SEM (*p < 0.05, **p < 0.01, ***p < 0.001, and ****p < 0.0001). For simple comparisons, Student’s *t*-tests was used. For multiple comparisons, analysis of variance (ANOVA) followed by Tukey’s multiple comparisons tests as the post hoc analysis were performed. To address sex as a biological variable in the 5xFAD mouse model secondary analysis by two-way ANOVA with sex as a between-subjects factor and treatment group as a within-subjects factor followed by post hoc analysis. All samples or animals were included in the statistical analysis unless otherwise specified.

## Results

### Regulation of microglial PPARα and TFEB signaling by KDS-5104

**Fig. 1:**
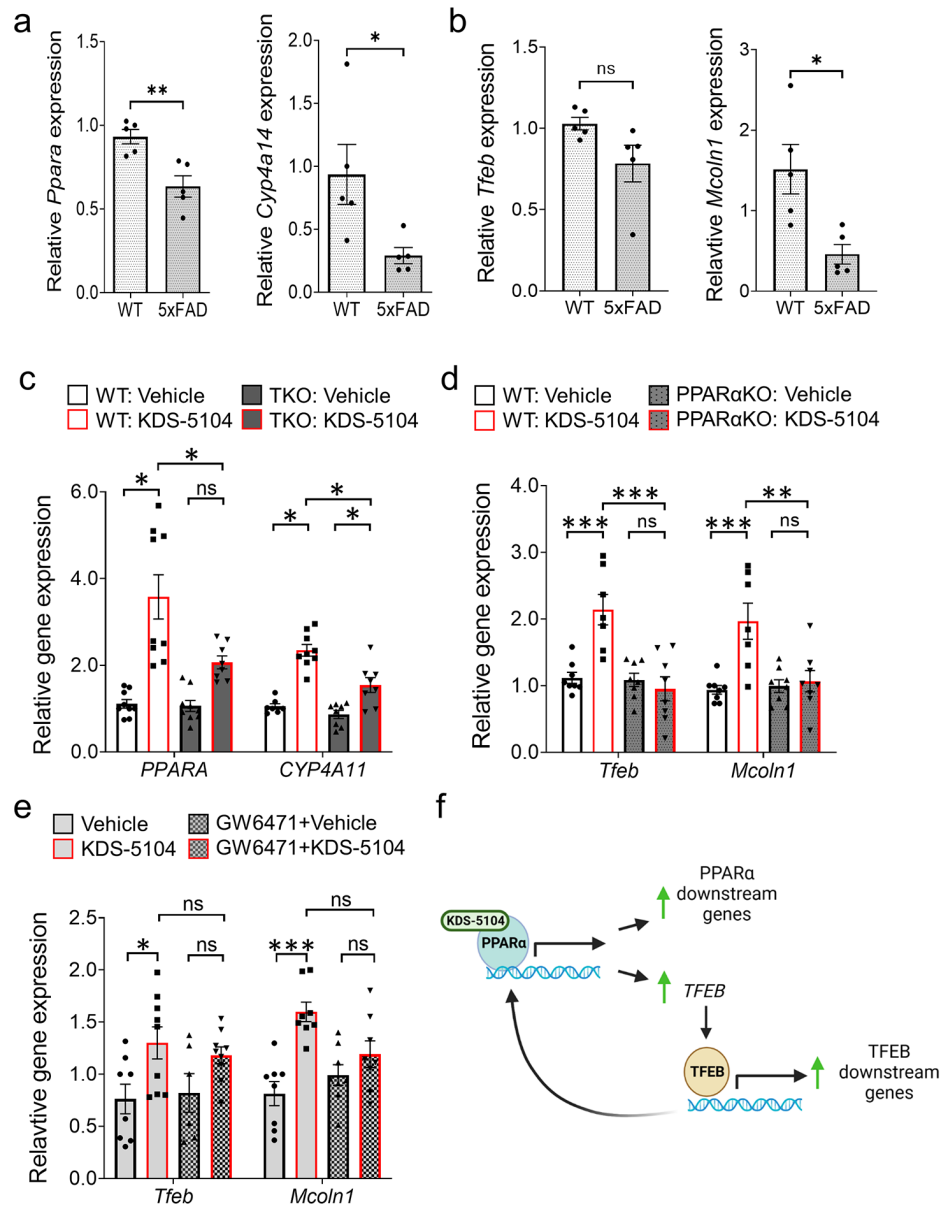
KDS-5104 activates PPARα and TFEB signaling in microglial cells. **(a)** qPCR analysis of expression of *Ppara* and downstream target *Cyp4a14* in sorted microglia isolated from brains of 9-month-old 5xFAD and WT littermates *(n = 5/group)*. **(b)** qPCR analysis of *Tfeb* and downstream target *Mcoln1* of the same samples as a) *(n = 5/group)*. **c.** qPCR analysis of *PPARA* and *CYP4A11* expression in KDS-5104 treated WT and TKO HeLa cells *(n = 9/condition)*. **d.** qPCR analysis of *Tfeb* and *Mcoln1* in WT and PPARαKO primary microglial cultures *(n = 8/condition)*. **e.** qPCR analysis of *Tfeb* and *Mcoln1* in vehicle or KDS-5104 treated BV2 cells with or without pretreatment with PPARα antagonist GW6471 *(n = 8/condition)*. **f.** Model of KDS-5104 action of PPARα activation and TFEB signaling. For all panels, data are presented as mean ± SEM. ns: non-significant, *p < 0.05, **p < 0.01, ***p < 0.001 by 2-sided *t* tests (a, b) or one way ANOVA with Tukey’s multiple comparisons tests as the post hoc analysis **(c-e)**

Since OEA was identified as a pro-longevity compound whose levels were dysregulated in AD plasma and CSF samples [3, 4], we first evaluated whether this is associated with altered expression of PPARα and its activity in aging mouse brains and postmortem AD brain samples, using its downstream target cytochrome P450 family 4 subfamily A (CYP4A) as a readout. Quantitative real-time PCR (qPCR) analysis showed that the expression of *Ppara* and *Cyp4a14* were decreased in 22-month-old mouse brains compared to 4 months (Fig. S1a). Similar reductions of their human counterparts *PPARA* and *CYP4A11* were also observed in postmortem AD brains (Fig. S1b), as well as PPARα protein levels by Western blotting (Fig. S1c & d). While levels of PPARα and its downstream target were not significantly changed in bulk brain samples of 5xFAD mice (Fig. S2a-c), analysis of FACS-isolated microglia identified a reduction of *Ppara* and *Cyp4a14* mRNA levels in 9-month-old 5xFAD mice compared to their WT littermates (Fig. 1a). This was corroborated by unbiased RNA sequencing of microglia isolated from the *APP*^NLGF^ knock-in (APP-KI) mouse model [36], which demonstrated a significant reduction of *Ppara* expression in APP-KI microglia compared to WT controls (Fig. S2d). Interestingly, analysis of TFEB and its downstream target MCOLN1 revealed similar changes. Specifically, while the expression of *Tfeb* and *Mcoln1* were indistinguishable in WT and 5xFAD bulk brain samples (Fig. S2e), their levels were trending (*Tfeb*) or significantly (*Mcoln1*) reduced in purified microglia of 5xFAD mice compared to WT controls (Fig. 1b). These results indicate reduced PPARα pathway associated with Aβ pathology, particularly in microglia, and a possible interaction between the PPARα and TFEB signaling pathways.

To decipher the relationship between PPARα and TFEB, we evaluated the effect of OEA, using its stable analog KDS-5104 (Fig. S3a), on PPARα in TFEB/TFE3/MITF triple knockout (TKO) HeLa cells [30], and conversely, TFEB activity in primary microglia cultured from *Ppara* knockout (PPARαKO) mice [37], given the specific reduction of *Ppara* in microglia of 5xFAD mice. Treating the WT microglial cultures with KDS-5104 showed dose-dependent increases of *Ppara* and *Cyp4a14* (Fig. S3b), as well as *Tfeb* and *Mcoln1* (Fig. S3c). Treating the WT and TKO cells with KDS-5104 resulted in increased *PPARA* and *CYP4A11* expressions, however, the degree of activation was substantially lower in TKO cells (Fig. 1c), suggesting that OEA/KDS-5104 could act on the PPARα pathway in the absence of TFEB although the maximal activation may require TFEB. In contrary, treating the primary microglial cultures from WT and PPARαKO mice with KDS-5104 showed that, while both *Tfeb* and *Mcoln1* expression were upregulated by KDS-5104, this response was blunted in PPARαKO cultures (Fig. 1d). Similar results were also obtained when BV2 cells were treated with the PPARα antagonist GW6471 (Fig. 1e). Together these results support a model whereby KDS-5104 acts directly on PPARα to upregulate the expression of its downstream genes and indirectly on TFEB transcription and signaling through PPARα, which in turn feedback to augment the PPARα activity (Fig. 1f).

### KDS-5104 activates TFEB lysosomal pathway independent of mTORC1

**Fig. 2:**
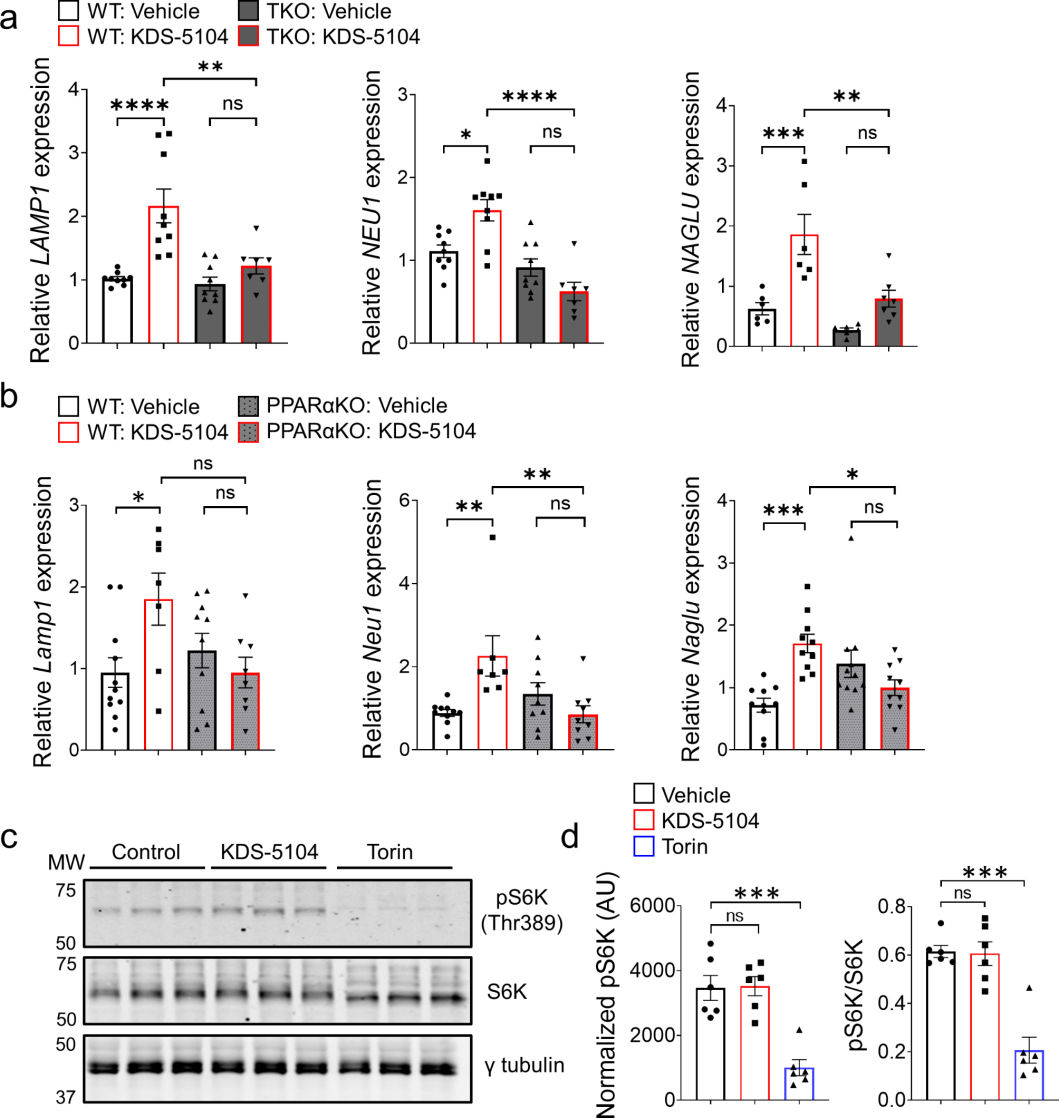
KDS-5104 increases TFEB lysosomal genes independent of mTORC1. **(a)** qPCR analysis of lysosome enzyme genes *LAMP1*, *NEU1*, and *NAGLU* in vehicle or KDS-5104 treated WT and TKO cells *(n = 8/condition)*. **(b)** qPCR analysis of *Lamp1, Neu1, Naglu* in vehicle or KDS-5104 treated WT and PPARαKO primary microglia cultures *(n = 10/condition)*. **(c)** Western blot analysis of total and phosphorylated S6K (pS6K) levels in primary microglial cultures treated with vehicle (Control), KDS-5104 (10 µM) or Torin (500 nM). γ-tubulin was used as a loading control. **(d)** Quantification of pS6k expression level normalized to γ-tubulin (left) and the ratio of pS6k to total S6k (right) *(n = 6/condition)*. AU: artificial unit. For all panels, data are presented as mean ± SEM. ns: non-significant, *p < 0.05, **p < 0.01, ***p < 0.001, ****p < 0.0001. One way ANOVA with Tukey’s multiple comparisons tests as the post hoc analysis

TFEB is known to be a master regulator of lysosomal biogenesis by activating multiple lysosomal genes [38, 39]. We thus aimed to determine if KDS-5104 induces changes of lysosomal genes through PPARα and subsequent TFEB activation. Indeed, qPCR analysis documented an increase in the expression of several TFEB lysosomal targets including *LAMP1*, neuraminidase 1 (*NEU1*), and alpha-N-acetylglucosamindase (*NAGLU)* in KDS-5104 treated WT HeLa cells, which were blunted in TKO cells (Fig. 2a). This is also the case when KDS-5104 were applied to WT, but not PPARαKO, primary microglial cultures (Fig. 2b), consistent with the idea that PPARα is necessary for KDS-5104 induced TFEB activation.

It is well-established that TFEB activity is tightly regulated by mTORC1 through TFEB phosphorylation and nuclear translation [40, 41]. To assess a possible role of mTORC1 in KDS-5104 induced TFEB activation, we measured the levels of the mTORC1 target, phospho-p70 S6 kinase (pS6K) at Thr 389 site [42], upon treating the HeLa cells with KDS-5104 or mTORC1 inhibitor Torin. A drastic reduction of pS6K were observed when the cells were treated with Torin (Fig. 2c). In contrast, we found no changes of pS6K by KDS-5104 treatment (Fig. 2c and quantified in 2d), suggesting that KDS-5104 does not influence mTORC1 activity. Similarly, KDS-5104 had no effect on Akt activity, which has been reported as an mTORC1-independent regulator of TFEB [43] (Fig. S3d & e). These results provide support that KDS-5104 activates TFEB through PPARα-dependent but mTORC1-independent mechanisms.

### KDS-5104-PPARα signaling promotes lysosomal biogenesis

**Fig. 3:**
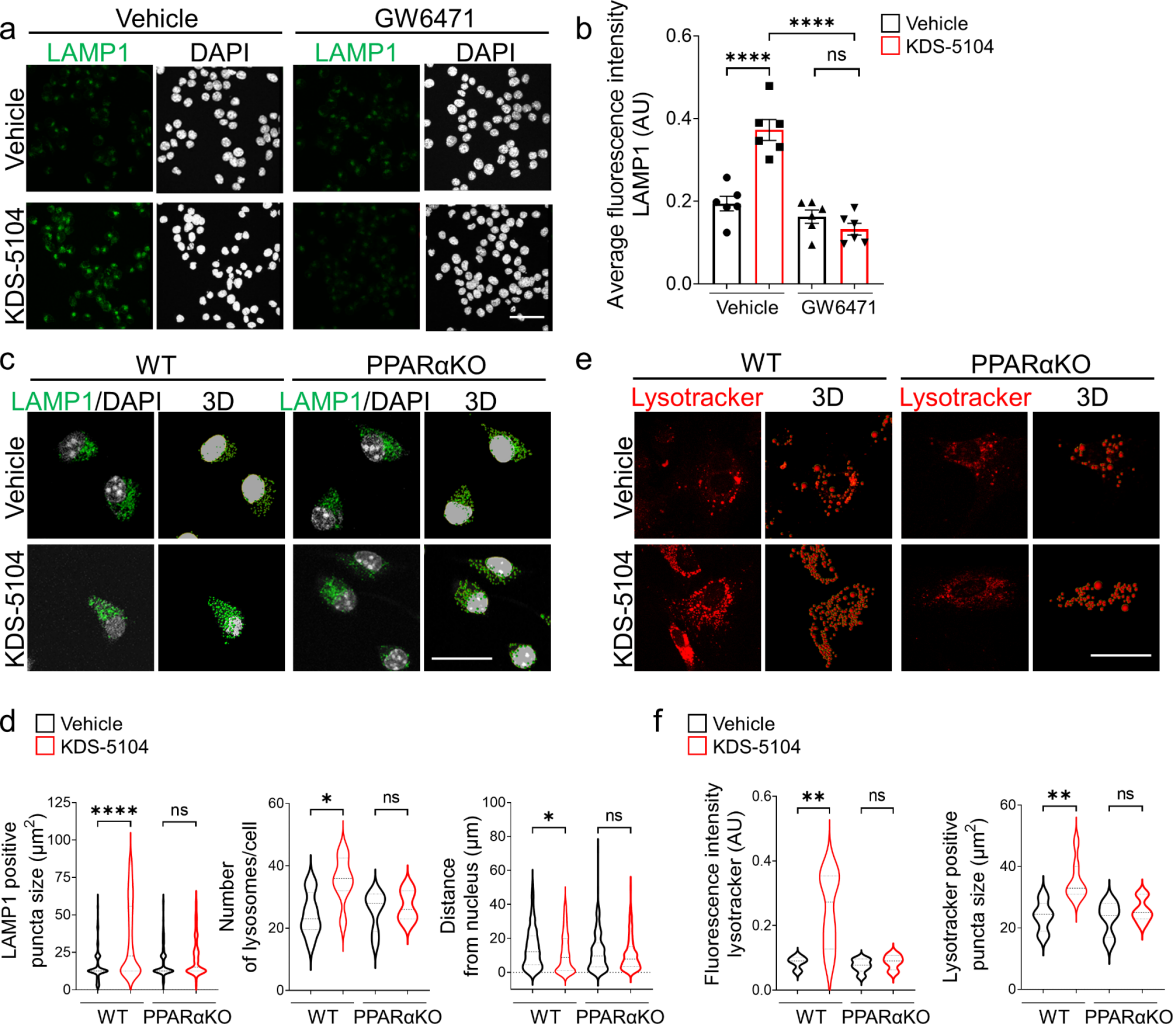
KDS-5104 increases lysosomal biogenesis in a PPARα dependent manner. **(a)** Representative LAMP1 immunofluorescence images of vehicle or KDS-5104 treated BV2 cells with or without GW6471 pretreatment. **(b)** Quantification of LAMP1 fluorescent intensity *(n = 6/condition)*. **(c)** Representative images of LAMP1 (green) and DAPI (white) staining and corresponding 3D renderings of primary WT and PPARαKO microglia cultures treated with vehicle or 10 µM KDS-5104 for 8 h. **(d)** Quantification of lysosome size, number of lysosomes per cell and distance of lysosomes from the nucleus. **(e)** Representative images of lysotracker positive lysosomes and 3D renderings of primary WT and PPARαKO microglial cultures treated with vehicle or 10 µM KDS-5104 for 8 h. **(f)** Quantification of lysotracker fluorescence intensity and number of lysotracker + lysosomes per cell. Approximately 25 cells obtained from a minimum of 3 wells per group were analyzed. AU: artificial unit. Scale bars: 200 μm in (a) and 100 μm in (c, e). For all panels, data are presented as mean ± SEM. ns: non-significant, *p < 0.05, **p < 0.01, ****p < 0.0001. One way ANOVA with Tukey’s multiple comparisons tests as the post hoc analysis

We next asked whether increased expression of TFEB lysosomal genes by OEA/KDS-5104 is associated with higher lysosomal activity and whether such an effect is PPARα dependent. We treated the BV2 cells with KDS-5104 and performed immunofluorescence staining with an anti-LAMP1 antibody to mark the lysosome (Fig. 3a). KDS-5104 treatment led to higher LAMP1 intensity, indicating increased lysosomal content. Co-treatment with the PPARα antagonist GW6471 abolished the KDS-5104 effects (Fig. 3a & b), consistent with the notion that KDS-5104 promotes lysosomal activity through the PPARα-TFEB axis. To provide additional support, we generated primary microglial cultures from PPARαKO and littermate WT controls and used Imaris imaging software to analyze the properties of lysosomes visualized by LAMP1 immunofluorescence (Fig. 3c). In KDS-5104 treated WT microglia, both the lysosomal size and lysosomal number were higher compared to vehicle treated controls (Fig. 3d), indicating an increased lysosomal activity and biogenesis. Further analysis showed that the lysosomes in KDS-5104 treated cells were in closer proximity to the nucleus, further supporting increased lysosomal activity (Fig. 3d). Consistent with the PPARα dependent mechanism, PPARαKO microglia treated with KDS-5104 did not display an increase in lysosome size, number, or altered distance to nucleus compared to vehicle treated PPARαKO microglia (Fig. 3d). Lastly, we used lysotracker to measure the lysosomal activity (Fig. 3e). The KDS-5104 treated WT microglia showed an increase in the lysotracker intensity and lysotracker-positive puncta size, and these effects were abolished in PPARαKO microglia (Fig. 3f). Together, the increase in lysosome size, number, acidity and proximity to nucleus by KDS-5104 treatment in WT but not PPARαKO microglia provide strong support that OEA/KDS-5104 promotes lysosomal activity and biogenesis in a PPARα dependent manner.

### KDS-5104 promotes microglial Aβ phagocytosis through PPARα-CD36 axis

**Fig. 4:**
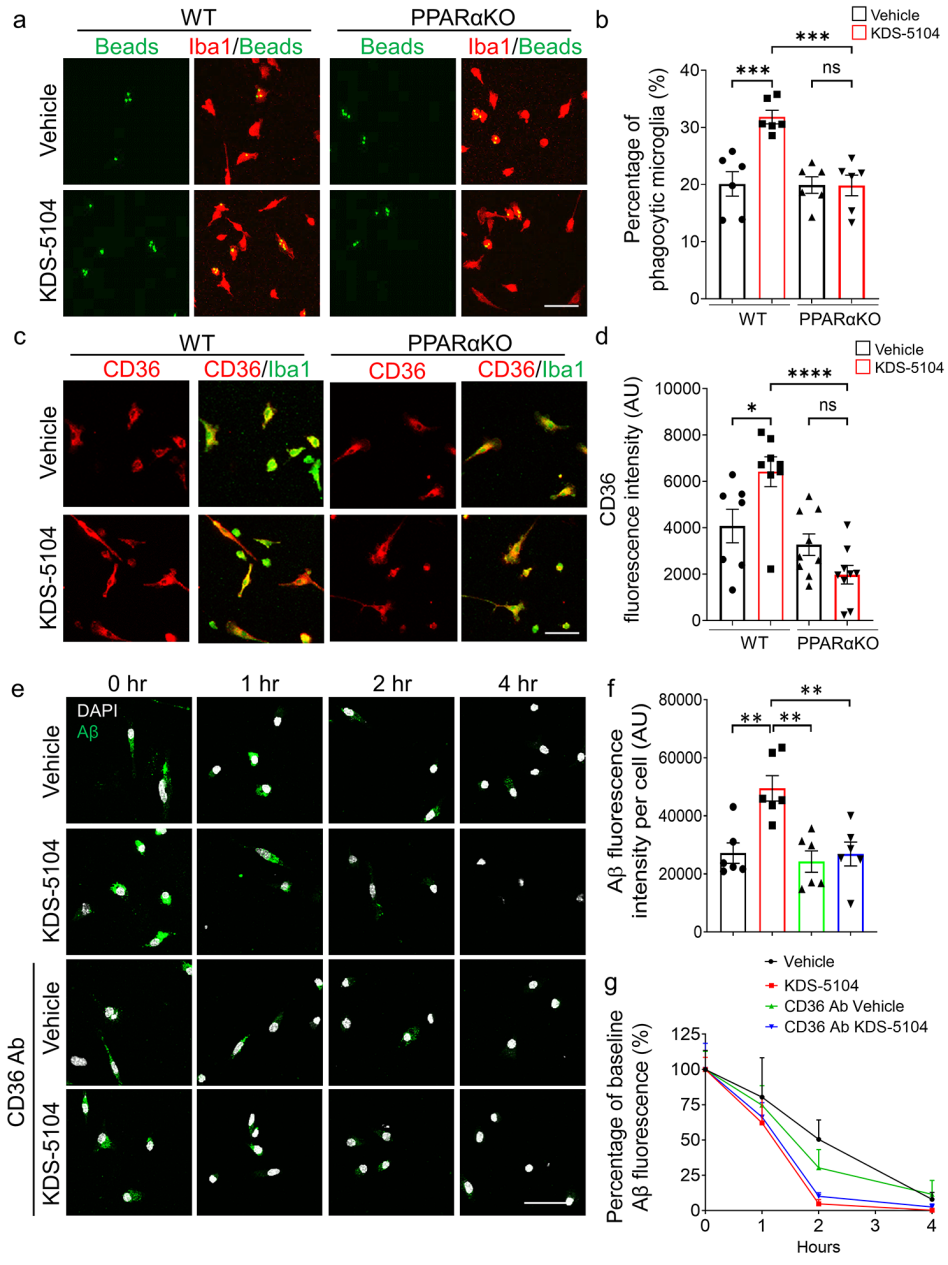
KDS-5104-PPARα pathway promotes phagocytosis and Aβ clearance. **(a)** Representative images of fluorescent beads (green) uptake in Iba1 (red) positive WT and PPARαKO primary microglia cultures treated with vehicle or 10 µM KDS-5104 for 8 h. **(b)** Quantification of the percentage of microglia positive cells with fluorescent beads *(n = 6/condition)*. **(c)** Representative images of CD36 (green) and Iba1 (red) staining in primary WT and PPARαKO microglia cultures treated with vehicle or KDS-5104. **(d)** Quantification of CD36 fluorescence intensity per cell of ~ 100 cells *(n = 7–9/condition*). **(e)** Time course of fluorescent labeled Aβ (green) and DAPI (white) in vehicle or KDS-5104 treated WT microglial cultures with or without pretreatment with the CD36 neutralizing antibody. **(f)** Analysis of Aβ fluorescence intensity per cell of ~ 100 cells at time of zero of Aβ removal *(n = 6/condition*). **(g)** Quantification of the percentage of baseline fluorescence remaining 1 h, 2 h, and 4 h after the removal of Aβ *(n = 6/condition*). Scale bars: 100 μm. For all panels, data are presented as mean ± SEM. ns: non-significant, *p < 0.05, **p < 0.01, ***p < 0.001, ****p < 0.0001. One way ANOVA with Tukey’s multiple comparisons tests as the post hoc analysis

One of the main functions of microglia is to mediate phagocytosis of extracellular materials and clearance by the lysosome. The above experiments established a role of OEA in regulating lysosomal activity. We next evaluated its effect in phagocytosis. Treating the primary microglial cultures with KDS-5104 resulted in increases of multiple phagocytosis related genes including *Fcer1g*, *Fcgr2b*, *Trem2, Cd36* (Fig. S4). Supporting a functional role of the phagocytic gene expression, analysis of fluorescently labelled beads uptake showed that KDS-5104 treated WT microglia displayed a higher internalization, but this effect was abolished when PPARα is inactivated (Fig. 4a & b). Among the phagocytic markers that were upregulated by KDS-5104, CD36 is known to be an Aβ scavenger receptor, and a direct downstream gene activated by PPARα [44]. Consistent with the RNA expression, immunofluorescence staining showed a PPARα dependent increase in CD36 protein expression in KDS-5104 treated microglia (Fig. 4c & d), which is associated with increased engulfment of FITC labelled Aβ (Fig. 4e & f, Vehicle vs. KDS-5104 at 0 h). In agreement with a direct functional role of CD36 in Aβ phagocytosis, increased Aβ uptake by KDS-5104 was blocked by pretreating the cultures with a CD36 neutralizing antibody (Fig. 4e & f, CD36 Ab Vehicle vs. CD36 Aβ KDS-5104 at 0 h). Analysis of Aβ degradation by measuring the percentage of Aβ remaining at various time points post Aβ uptake showed that KDS-5104 treated microglia had a significantly reduced percentage of Aβ remaining compared to the vehicle treated microglia (Fig. 4g, Vehicle vs. KDS-5104), and these effects are similar with or without anti-CD36 antibody treatment (Fig. 4g, KDS-5104 and CD36 Ab KDS-5104), suggesting that CD36 mediates Aβ uptake but not degradation. Altogether, the results combined support a model by which PPARα-CD36 signaling regulates Aβ phagocytosis while PPARα-TFEB interaction promotes Aβ lysosomal degradation.

### KDS-5104 decreases LPS-induced inflammation and lipid droplet formation

**Fig. 5:**
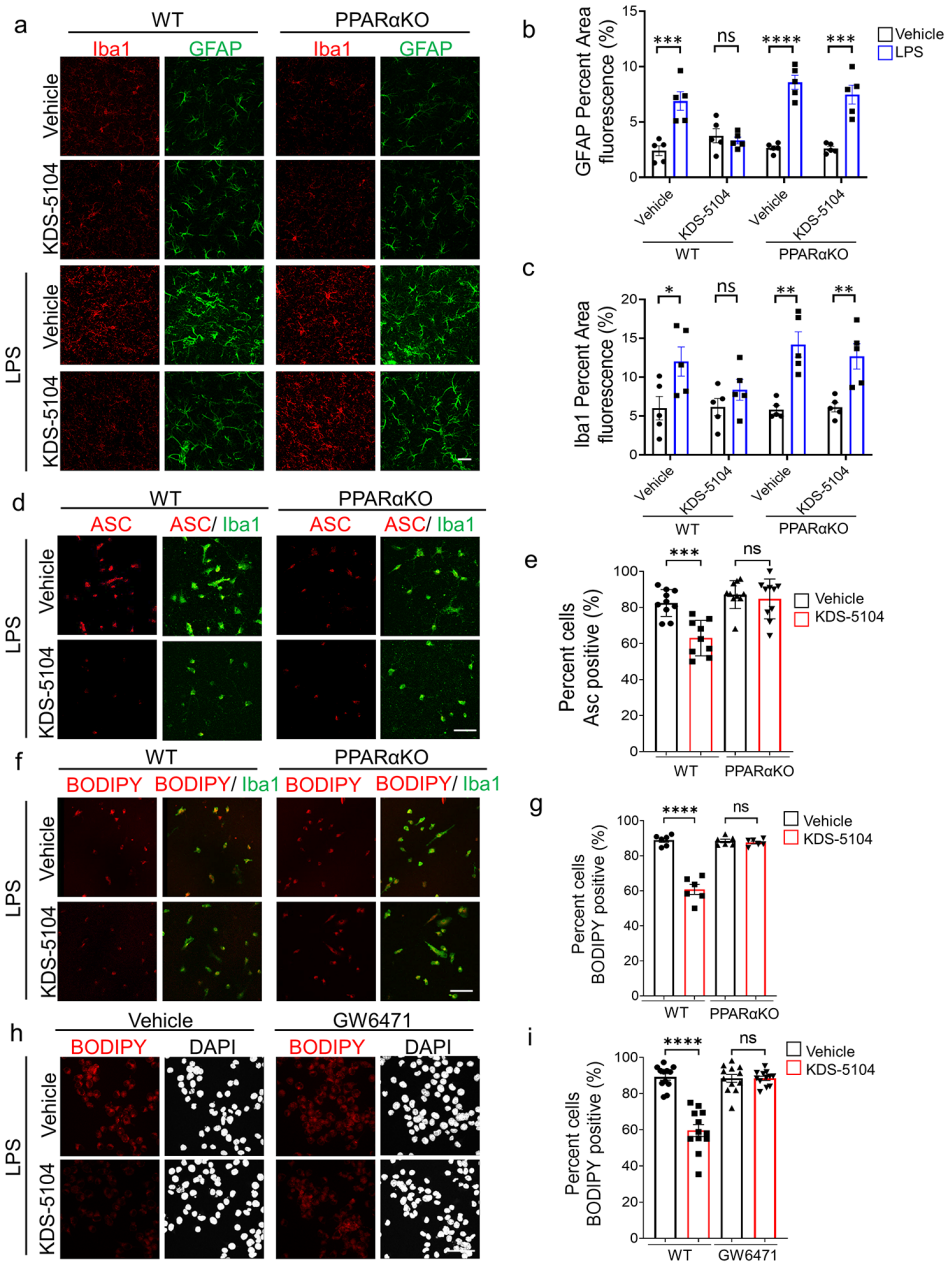
KDS-5104 reduces LPS-induced inflammation and lipid droplet formation via PPARα activation. **(a)** Representative images of Iba1 and GFAP from 4-month-old WT and PPARαKO mice pretreated with vehicle or 10 mg/kg KDS-5104 (i.p.) for 24 h, followed by a co-treatment of LPS (2 mg/kg, i.p.) and KDS-5104 (10 mg/kg, i.p.) for 18 h. **(b)** Quantification of GFAP fluorescent area *(n = 5 mice/group)*. **(c)** Quantification of Iba1 fluorescent area *(n = 5 mice/group)*. **(d)** Representative images of ASC speck (red) in primary WT and PPARαKO microglia cultures pretreated with vehicle or KDS-5104 for 18 h before addition of LPS. **(e)** Quantification of percentage of ASC positive cells *(n = 9/group)*. **(f)** Same as (d) except BODIPY + lipid droplet was imaged. **(g)** Quantification of percentage of BODIPY positive cells *(n = 6/group)*. **(h)** Representative images of lipid droplet formation by BODIPY (red) and DAPI (white) in vehicle or KDS-5104 pretreated and LPS induced BV2 cells without or with GW6471. **(i)** Quantification of percentage of BODIPY cells *(n = 12/group)*. Scale bar: 100 μm. For all panels, data are presented as mean ± SEM. ns: non- significant, *p < 0.05, **p < 0.01, ***p < 0.001, ****p < 0.0001. One way ANOVA with Tukey’s multiple comparisons tests as the post hoc analysis

Given the reported anti-inflammatory effect of PPARα [45], we next tested the role of KDS-5104 in LPS-induced neuroinflammation. WT and PPARαKO mice received a pre-treatment of KDS-5104 (10 mg/kg) for 24 h, followed by a co-treatment of LPS (2 mg/kg) and KDS-5104 for 18 h. Analysis of Iba1 and GFAP immunoreactivities showed increased levels in WT and PPARαKO mice upon LPS treatment (Fig. 5a). KDS-5104 attenuated this effect in WT mice but not in LPS treated PPARαKO mice (Fig. 5a-c). LPS also induced ASC specks, an indicator of inflammasome activation, in both WT and PPARαKO microglia cultures (Fig. 5d). KDS-5104 treatment resulted in reduced ASC specks in WT cultures. This suppression was attenuated in PPARαKO microglia (Fig. 5d & e).

Besides the regulation of inflammatory processes, PPARα plays a potent role in fatty acid oxidation and lipid homeostasis [46]. We thus assessed the effect of the KDS-5104-PPARα axis on LPS-induced lipid droplet accumulation. We found that KDS-5104 treatment effectively reduced lipid droplets induced by LPS in WT, but not in PPARαKO microglial cultures (Fig. 5f & g). In support of the genetic knockout, PPARα antagonist GW6471 also blunted KDS-5104 effects on inflammation induced lipid droplet formation in BV2 cells (Fig. 5h & i). Together, these results establish the beneficial effect of KDS-5104 in suppression of inflammation and lipid droplet accumulation and these effects are PPARα dependent.

### KDS-5104 treatment restores lipid dysregulation in 5xFAD mice

Emerging evidence suggests that lipid dysregulation is a key event in the development of AD [47]. Specifically, lipid profile alterations have been identified in microglia with reduced phagocytosis capabilities which can lead to aberrant Aβ accumulation [48, 49]. After establishing that KDS-5104-PPARα axis plays a pivotal role in regulating LPS-induced lipid droplet formation, we next aimed to determine the effect of KDS-5104 in AD mouse models.

We first established a proper dosing to ensure efficacy but no adverse effects, particularly body weight given the known satiety effect of OEA [5, 6]. A subchronic regime was performed where WT mice received a dose of KDS-5104 at either 10 mg/kg or 50 mg/kg every other day for 3 weeks. The 50 mg/kg treatment group had a significant decrease in body weight during the three-week treatment, however, at 10 mg/kg, the body weight was maintained similar to vehicle treatment in both males and females (Fig. S5a & b). Regardless, no appreciable differences in animal behavior including rotarod and grip strength were observed in either treatment groups (Fig. S5c & d). qPCR analysis of the cortex (Fig. S4e) and the liver (Fig. S4f) showed increased expression of PPARα and TFEB pathway genes in both tissues. These results suggest that KDS-5104 can upregulate PPARα and TFEB in both peripheral tissues and the brain and that 10 mg/kg is sufficient to achieve in vivo efficacy without overt side effects.

**Fig. 6:**
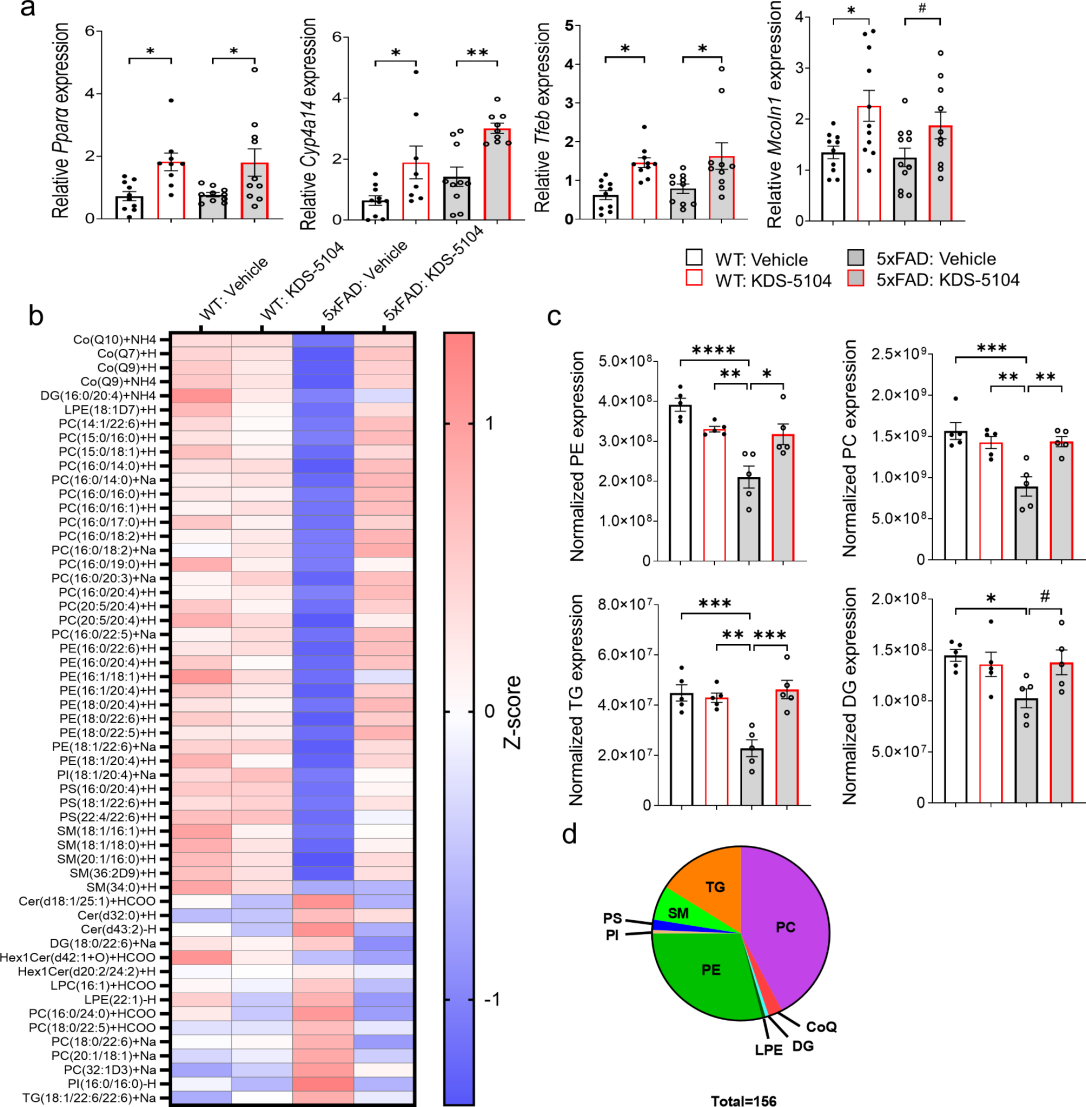
Reversed lipid dysregulation in 5xFAD mice with KDS-5104 treatment. **(a)** qPCR analysis of *Ppara*, *Cyp4a14, Tfeb* and *Mcoln1* in 4-month-old WT and 5xFAD mice treated with vehicle or KDS-5104 for 2 months *(n = 9/group)*. **(b)** Heatmap representing z scores of top 55 dysregulated lipid species in 5xFAD mice identified by lipidomic analysis of 4-month-old WT and 5xFAD mice treated with vehicle or KDS-5104. **(c)** Normalized levels of the lipid species per lipid class *(n = 5 mice/group)*. PE: phosphatidylethanolamine, PC: phosphatidycholine, PS: phosphatidylserine, DG: diglycerides, and TG: trigylcerides (TG). **(d)** Pie chart depicting the classes of the 156 lipids identified that are dysregulated in 5xFAD mice compared to WT and rescued with KDS-5104 treatment. CoQ: Coenzyme Q10, LPE: Lysophosphatidylethanolamine, SM: Sphingomyelin, and PI: Phosphatidylinositol. For all panels, data are presented as mean ± SEM. ^#^*p* = 0.2 in (a) and 0.1 in (c), *p < 0.05, **p < 0.01, ***p < 0.001, ****p < 0.0001. One way ANOVA with Tukey’s multiple comparisons tests as the post hoc analysis

We next treated WT and 5xFAD mice with KDS-5104 (10 mg/kg, i.p.) or vehicle starting at 2 months of age when the 5xFAD mice begin to develop Aβ pathology for a total of 2 months. By the conclusion of the study at age of 4 months, 5xFAD mice display ample Aβ pathology, neuroinflammation, metabolic dysfunction and behavioral changes [29, 50–52]. Targeting this early age thus allows us to investigate the potential of KDS-5104 to modulate early biochemical and functional alterations that drive AD pathogenesis at late stages. Consistent with the subchronic dosing, we found an increase in the expression of *Pparα*, *Cyp4a14, Tfeb and Mcoln1* in KDS-5104 treated WT and 5xFAD mice (Fig. 6a).

Given the well-known function of PPARα in lipid regulation, we performed untargeted lipidomic analysis of bulk cortical tissue from vehicle or KDS-5104 treated WT and 5xFAD mice and identified a total of 939 distinct lipid species. We generated a heatmap using the Z score of the top 55 lipid species with statistically significant alterations in 5xFAD compared to WT mice (Fig. 6b). We further calculated the total abundance of major lipid classes: phosphatidylethanolamine (PE), phosphatidycholine (PC), diglycerides (DG) and trigylcerides (TG). All except SM showed significant reductions in vehicle treated 5xFAD mice compared to WT controls (Fig. 6c), possibly reflects the overall metabolic dysregulation. KDS-5104 treatment resulted in significant increases of PE, PC and TG levels and trended upwards (*p* = 0.1) in DG (Fig. 6c). In total, we identified 156 lipids that were dysregulated in 5xFAD mice compared to WT that was recovered by KDS-5104 treatment (Fig. 6d, Supplementary Table 2).

### Attenuation of Aβ pathology and cognitive deficit by KDS-5104 treatment

**Fig. 7:**
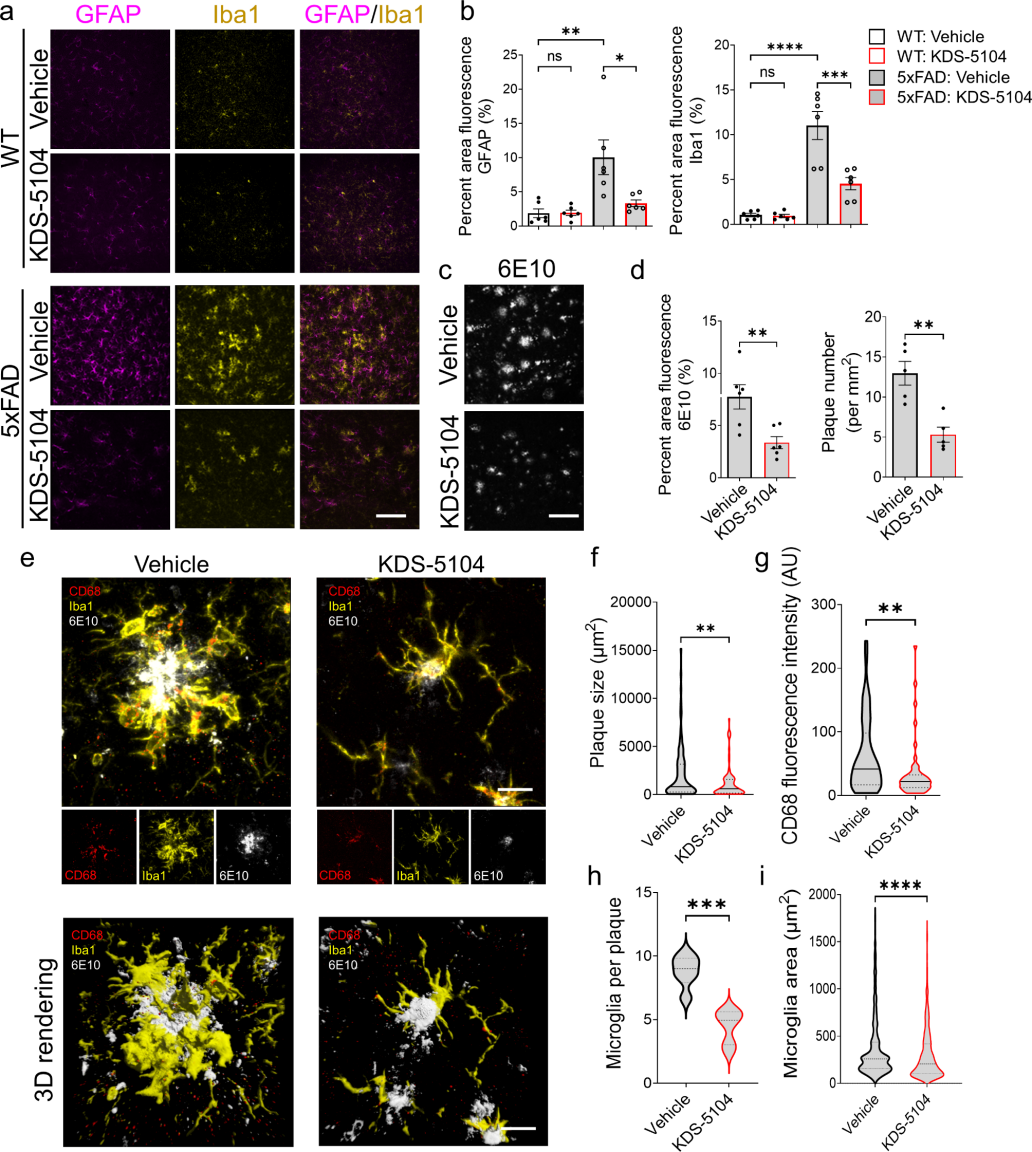
Reduced reactive gliosis and Aβ pathology in 5xFAD mice treated with KDS-5104. **(a)** Representative images of GFAP (magenta) and Iba1 (yellow) co-staining from the hippocampal sections of 4-month-old 5xFAD and WT mice treated with vehicle or 10 mg/kg KDS-5104 for two months. Scale bar:. **(b)** Quantification of Iba1 and GFAP area fluorescence *(n = 5–6 mice/group)*. **(c)** Representative images of 6E10 (white) of hippocampal sections of 4-month-old 5xFAD treated with vehicle or KDS-5104. **(d)** Quantification of 6E10 positive area and plaque number *(n = 5–6 mice/group)*. **(e)** Representative images of CD68 (red), Iba1 (yellow) and 6E10 (white) co-staining and 3D rendering created by Imaris imaging software. **f-h.** Quantification of plaque size **(f)**, CD68 fluorescent intensity **(g)**, microglia per plaque **(h)** and microglia area **(i)***(n = 5–6 mice/group)*. AU: artificial unit. Scale bar: 100 μm in **(a)** and **(c)** and 10 μm in **(e)**. For all panels, data are presented as mean ± SEM. *p < 0.05, **p < 0.01, ***p < 0.001, ****p < 0.0001 by one way ANOVA with Tukey’s multiple comparisons tests as the post hoc analysis (panel **b**) or 2-sided *t*-tests (panels **d** and **f-i**)

We next examined the AD neuropathology in the 5xFAD mice treated with KDS-5104. Immunofluorescence staining of GFAP and Iba1 showed similar patterns in vehicle or KDS-5104 treated WT mice (Fig. 7a & b). As expected, both GFAP and Iba1 immunoreactivities were increased in 4-month-old 5xFAD mice, and these were drastically reduced by KDS-5104 treatment (Fig. 7a & b). These were correlated with elevated expression of disease associated microglia (DAM) marker genes in 5xFAD mice and their suppressions by KDS-5104 treatment (Fig. S6a). As astrocyte reactivity can be induced by microglia secreted IL-1α, TNFα and C1q [53]. We performed qPCR analysis of these factors, which showed increased expressions in 5xFAD mice and dampened by KDS-5104 treatment (Fig. S6b). Thus the reactive astrogliosis marked by GFAP may be downstream of heightened microglia activation.

Analysis of Aβ pathology using the 6E10 antibody showed reduced Aβ immunointensities and plaque numbers in 5xFAD mice treated with KDS-5104 (Fig. 7c & d). We observed a reduced plaque size and number in 5xFAD mice treated with KDS-5104 compared to vehicle (Fig. 7d). Further characterization of microglia surrounding the plaques by co-staining with the phagocytic marker CD68 and by 3D rendering revealed reduced CD68 levels, plaque size and plaque associated microglia number and area in KDS-5104 treated 5xFAD (Fig. 7e-i). In agreement with our in vitro data on the increased CD36 expression and suppression of inflammation induced lipid droplet formation by KDS-5104, qPCR analysis documented reduced *Cd36* in bulk brain samples of 5xFAD mice that were rescued by KDS-5104 treatment (Fig. S6c). Immunostaining of the lipid droplet surface protein Perilipin 2 (Plin2) in brain sections of vehicle and KDS-5104 treated 5xFAD mice revealed reduced Plin2 staining in Iba1 positive microglia surrounding Aβ plaques by KDS-5104 treatment (Fig. S6d & e).

Since PPARα has been reported to regulate ADAM10 activity and α-secretase processing of APP [15], we examined levels of full-length APP, its C-terminal fragments (APP-CTF), and ADAM10 by Western blotting. We observed no appreciable differences between KDS-5104 and vehicle treated 5xFAD (Fig. S7). Thus, the reduction in Aβ pathology is unlikely due to changes in APP expression or processing.

**Fig. 8:**
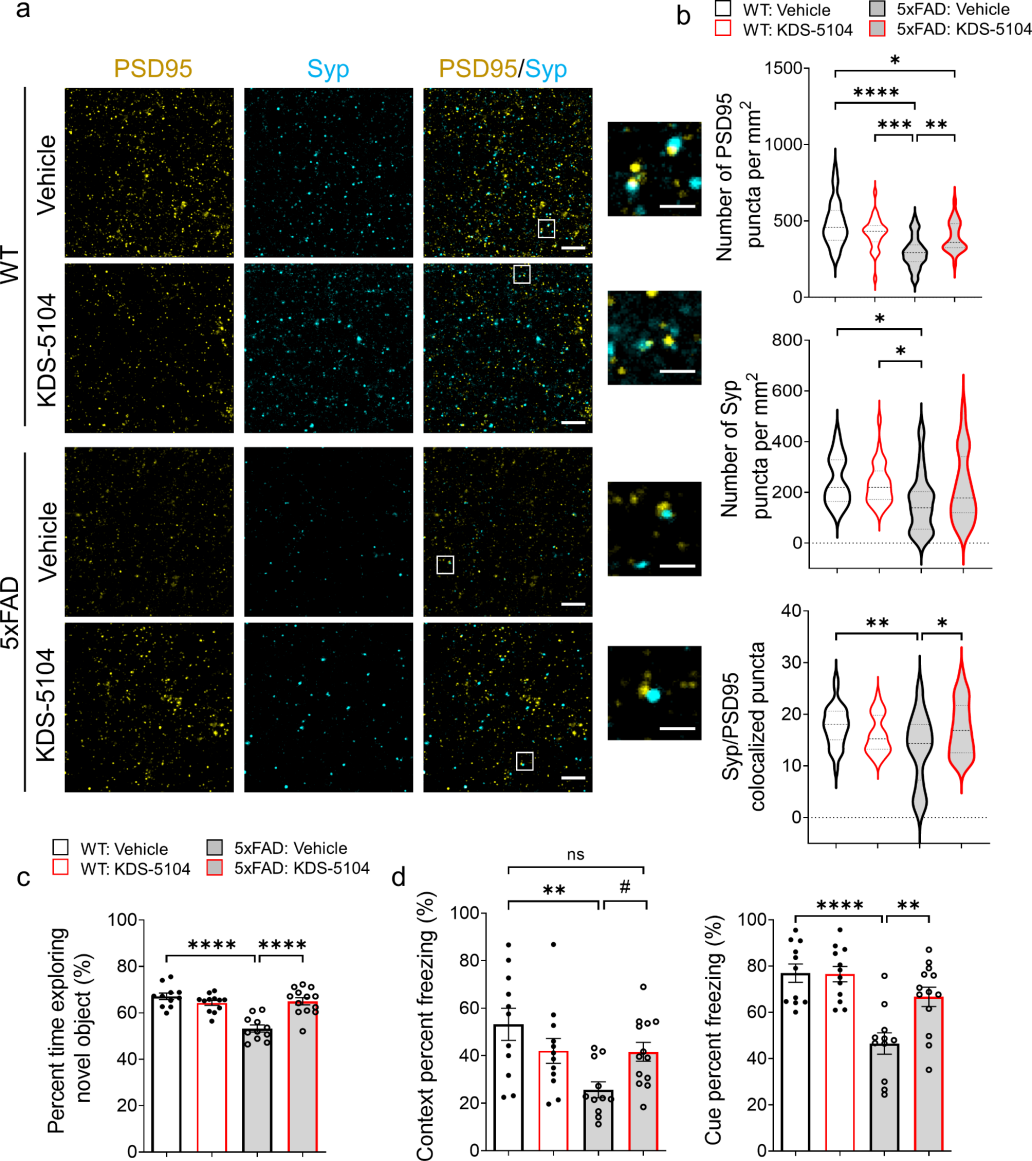
Improved synaptic properties and cognitive performance in 5xFAD mice treated with KDS-5104. **(a)** Representative images of co-staining of post-synaptic marker PSD95 and presynaptic marker synaptophysin (Syp) from hippocampal CA1 area of 4-month-old WT and 5xFAD mice treated with vehicle or 10 mg/kg KDS-5104. Inset showing enlarged view of Syp and PSD95 co-localized puncta. Scale bar: 100 μm; 25 μm zoom in. **(b)** Quantification of the number of PSD95, Syp and co-localized Syp and PSD95 puncta *(n = 6 mice/group)*. **(c)** Quantification of percent time exploring the novel object in NOR assay. **(d)** Freezing percentage due to contextual or cue testing in the fear conditioning paradigm *(n = 11–13 mice/group)*. For all panels, data are presented as mean ± SEM. ns: non- significant, ^#^*p* = 0.11, *p < 0.05, **p < 0.01, ***p < 0.001, ****p < 0.0001. One way ANOVA with Tukey’s multiple comparisons tests as the post hoc analysis

Having established reduced Aβ pathology and reactive gliosis by KDS-5104 treatment, we next assessed its effect on synaptic and behavioral phenotypes. High resolution imaging of the presynaptic protein, synaptophysin (Syp), and the postsynaptic protein, PSD95, revealed that the overall levels of Syp and PSD95 and colocalization of the pre- and post- synaptic puncta were significantly lower in 5xFAD mice compared to WT controls. Treatment with KDS-5104 led to increased pre- and post-synaptic markers as well as colocalized synaptic puncta (Fig. 8a & b). We further performed cognitive testing to evaluate the functional effect of KDS-5104 treatment. Like subchronic treatment, there was no differences observed in body mass changes between KDS-5104 and vehicle treated groups (Fig. S8a). General neurological assessment also revealed no group differences in rotarod (Fig. S8b) or grip strength (Fig. S8c), suggesting no changes in general mobility and motor function between the groups and further verifying the safety of the drug treatment regime. Vehicle treated 5xFAD mice exhibited an increase in distance travelled and total movement time in open field arena suggesting hyperactivity, phenotypes of which was reduced by KDS-5104 treatment (Fig. S8d & e). To assess hippocampal-dependent long-term recognition memory, we performed the novel object recognition test (NOR) by measuring the percentage of exploration time of a novel object following a training of two identical objects (Fig. 8c). The four groups did not exhibit object bias during the training phase (Fig. S8f). Vehicle treated 5xFAD mice explored the novel object 50% of the time, indicating a lack of memory of the novel object. However, KDS-5104 treatment resulted in an increased exploration time of the novel object comparable to the WT mice (Fig. 8c), indicating restored memory. We further performed the fear conditioning assay to test hippocampal dependent (contextual test) and independent (cued test) associative learning. Vehicle treated 5xFAD mice exhibited a decrease in freezing in both the context and cue test compared to WT mice (Fig. 8d). The 5xFAD mice treated with KDS-5104 displayed a significant increase in freezing in the cue test and trended upwards in the context test compared to vehicle treated 5xFAD (Fig. 8d). In addition, no significant difference was observed when WT mice and KDS-5104 treated 5xFAD were compared indicating an improvement in cognitive function (Fig. 8d). Examination of sex specific effect on Aβ pathology and behavior did not identify significant differences (Fig. S9). Overall, KDS-5104 treatment resulted in increased synaptic marker expression and improved cognitive performance in 5xFAD mice.

## Discussion

In the current study, we investigated the role of OEA, an endogenous lipid amide with pro-longevity properties, in regulating cellular signaling and lysosomal function in vitro and lipid metabolism and AD pathogenesis in vivo. Using its stable analog KDS-5104, we provide data to show that OEA/KDS-5104 promotes PPARα and TFEB signaling, leading to enhanced Aβ phagocytosis and lysosomal clearance, respectively, and suppression of LPS-induced lipid droplet accumulation and inflammasome activation in cultured microglial cells. These are associated with normalization of altered lipid profiles, reduction of reactive gliosis and Aβ pathology and improvement of synaptic density and cognitive function in 5xFAD mice. To our knowledge this is the first time that a functional effect of OEA/KDS-5104 in the brain has been reported. Mechanistically, we reveal a feedforward regulation of the PPARα-TFEB signaling axis and a novel mTORC1 independent activation of TFEB by OEA/KDS-5104, the latter offers potential to bypass the adverse effects associated with mTORC1-dependent TFEB activators such as rapamycin.

The two major functions of microglia are phagocytosis of extracellular materials followed by intracellular clearance and immune and inflammatory pathway regulation. Our in vitro studies demonstrate that OEA/KDS-5104 influences both processes. While we present evidence that CD36 and TFEB mediates microglial Aβ phagocytosis and clearance downstream of PPARα, these effects should not be limited to Aβ as CD36 is known to be involved in lipid sensing and TFEB is a master regulator of lysosomal function inclusive of lipid clearance. Enhancing lipid trafficking and clearance may lead to the suppression of LPS-induced lipid droplet accumulation and inflammasome activation. Alternatively, the OEA-PPARα pathway could function to directly suppress LPS induced changes, which in turn may lead to improved microglia phagocytosis and clearance. Overall, our results that KDS-5104 enhances phagocytosis and suppresses neuroinflammation and lipid droplet accumulation in vitro and in vivo are consistent with the finding that lipid droplet associated microglia are proinflammatory and phagocytosis deficient [49, 54].

We detected reduced PPARα pathway in postmortem human AD brains. While this observation is consistent with some published reports [55], others have implicated an inverse relationship between PPARα and APP and a reduction of PPARα in frontal cortices of AD brain tissues [56]. The inconsistencies could be due to confounding factors in the samples tested including but not limited to age, stage of disease progression, mixed pathology, region studied, and postmortem delays. Regardless, our results are to the most part in agreement with the beneficial effects of PPARα agonists in AD models reported by others [14–21]. Nevertheless, it is worth noting that, besides PPARα, OEA has been reported to bind to other receptors such as GPR119 [57], leading to peripheral regulation of lipid metabolism. Therefore, we cannot disregard the possibility of the involvement of PPARα independent mechanisms in the mediation of OEA/KDS-5104 effect.

OEA is well-known for its function in suppression of food intake and body weight gain, particularly under high-fat diet conditions. This has been suggested to be mediated by the peripheral sensory fibers and through dopamine signaling [58]. Although we did not observe an overt body weight difference between vehicle and KDS-5104 treated mice with the dose we administered (10 mg/kg), it is still possible that both mechanisms could contribute to the CNS effect we observed. Our result showing elevated PPARα and TFEB signaling in both the liver and the brain by KDS-5104 treatment is in keeping with this idea. Within the CNS, PPARα has been shown to be expressed and exert its effect in multiple cell types including neurons [15, 59] and astrocytes [18]. The specific impairment of the PPARα pathway in microglia of 5xFAD mice prompted us to focus our studies on microglia. However, it is likely that other cell types may also subject to OEA-PPARα regulation, the combination of which could result in the overall beneficial effect of KDS-5104 in 5xFAD mice, including bulk brain lipid profiles and Aβ associated pathologies. A microglial specific PPARα knockout will be helpful to address the cell type effect. In this regard, a recent paper revealed a role of astrocytic PPARα-TFEB and -LDLR pathway in Aβ uptake and clearance [18]. It is known that LDLR is expressed in microglia, thus could also mediate PPARα-dependent Aβ update. Nevertheless, our data that KDS-5104 induced Aβ phagocytosis was blocked when primary microglial cultures were treated with the CD36 neutralizing antibody supports a prominent role of this direct PPARα target in mediating KDS-5104 effect, either independently or by interacting with other Aβ and/or lipid receptors including LDLR and those upregulated by KDS-5104 [60]. Thus, while our studies demonstrate a beneficial role of OEA/KDS-5104 in rescuing AD neuropathology and cognition in 5xFAD mice, the cell type specific effects and precise molecular mechanisms require further investigation.

## Conclusions

Aging is the greatest risk factor for AD. Thus, agents that improve healthy aging may afford benefit in preventing or delaying AD. We present evidence that OEA may represent such a compound. Its reductions in the plasma and CSF of AD patients provide further disease relevance [4]. OEA augmentation offers several attractive features as a therapeutic strategy: First, it boosts an endogenous lipid signaling pathway; Second, it targets two molecules with therapeutic potentials, PPARα and TFEB, and the latter is mTORC1 independent; Lastly, OEA is relatively safe and is being marketed as a nutraceutical. Thus, our study calls for further development of OEA analogs as potential therapy for aging and AD.

### Electronic supplementary material

Below is the link to the electronic supplementary material.

Supplementary Material 1

## Data Availability

The datasets used and analyzed during the current study available from the corresponding author on reasonable request.

## List of abbreviations

Aβ: Amyloid beta
AD: Alzheimer’s disease
APP: Amyloid precursor protein
BODIPY: difluoroborondipyrromethene
CNS: Central nervous system
CoQ: Coenzyme Q10
DAM: Disease associated microglia
DAPI: 4’,6-diamidino-2-phenylindole
DG: Diacylglycerol
DMSO: Dimethylsulfoxide
GFAP: Glial fibrillary acidic protein
Iba1: Ionized calcium-binding adaptor molecule 1
LAMP1: Lysosomal-associated membrane protein 1
LDAM: Lipid droplet associated microglia
LOAD: Late onset Alzheimer’s disease
LPE: Lysophosphatidylethanolamine
LPS: Lipopolysaccharide
Naglu: N-acetyl-alpha-glucosaminidase
Neu1: Neuraminidase 1
NOR: Novel object recognition
MITF: Microphthalmia-associated transcription factor
mTORC1: Mammalian target of rapamycin complex 1
OEA: Oleoylethanolamide
PC: Phosphatidylcholine
PDL: Poly-D-Lysine
PE: Phosphatidylethanolamine
PI: Phosphatidylinositol
PPARα: Perxisome proliferator-activated receptor alpha
PPARα KO: Perxisome proliferator-activated receptor alpha knockout
PS: Phosphatidylserine
SM: Sphingomyelin
Syp: Synaptophysin
TFEB: Transcription factor EB
TFE3: Transcription factor binding to IGHM Enhancer 3
TKO: TFEB, TFE3, MITF triple knockout
TG: Triglyceride
WT: Wild-type

## References

[1] Querfurth HW, LaFerla FM (2010). Alzheimer’s Disease. N Engl J Med.

[2] Romero-Molina C, Garretti F, Andrews SJ, Marcora E, Goate AM (2022). Microglial efferocytosis: diving into the Alzheimer’s disease gene pool. Neuron.

[3] Folick A (2015). Lysosomal signaling molecules regulate longevity in *Caenorhabditis elegans*. Sci (1979).

[4] Borkowski K (2021). Association of plasma and CSF cytochrome P450, soluble epoxide hydrolase, and ethanolamide metabolism with Alzheimer’s disease. Alzheimers Res Ther.

[5] Fu J, Oveisi F, Gaetani S, Lin E, Piomelli D (2005). Oleoylethanolamide, an endogenous PPAR-α agonist, lowers body weight and hyperlipidemia in obese rats. Neuropharmacology.

[6] Fu J (2003). Oleylethanolamide regulates feeding and body weight through activation of the nuclear receptor PPAR-α. Nature.

[7] Settembre C, Ballabio A (2014). Cell metabolism: Autophagy transcribed. Nature.

[8] Braissant O, Foufelle F, Scotto C, Dauça M, Wahli W (1996). Differential expression of peroxisome proliferator-activated receptors (PPARs): tissue distribution of PPAR-alpha, -beta, and -gamma in the adult rat. Endocrinology.

[9] Aleshin S, Grabeklis S, Hanck T, Sergeeva M, Reiser G (2009). Peroxisome proliferator-activated receptor (PPAR)-gamma positively controls and PPARalpha negatively controls cyclooxygenase-2 expression in rat brain astrocytes through a convergence on PPARbeta/delta via mutual control of PPAR expression levels. Mol Pharmacol.

[10] Warden A (2016). Localization of PPAR isotypes in the adult mouse and human brain. Sci Rep.

[11] Aleshin S, Strokin M, Sergeeva M, Reiser G (2013). Peroxisome proliferator-activated receptor (PPAR)β/δ, a possible nexus of PPARα- and PPARγ-dependent molecular pathways in neurodegenerative diseases: review and novel hypotheses. Neurochem Int.

[12] Sáez-Orellana F, Octave J-N, Pierrot N (2020). Alzheimer’s Disease, a lipid story: involvement of peroxisome proliferator-activated receptor α. Cells.

[13] Wagner N, Wagner K-D (2020). The role of PPARs in Disease. Cells.

[14] Kummer MP (2015). Pan-PPAR modulation effectively protects APP/PS1 mice from amyloid deposition and cognitive deficits. Mol Neurobiol.

[15] Corbett GT, Gonzalez FJ, Pahan K (2015). Activation of peroxisome proliferator-activated receptor α stimulates ADAM10-mediated proteolysis of APP. Proc Natl Acad Sci.

[16] Chandra S, Roy A, Jana M, Pahan K (2019). Cinnamic acid activates PPARα to stimulate lysosomal biogenesis and lower amyloid plaque pathology in an Alzheimer’s disease mouse model. Neurobiol Dis.

[17] Luo R (2020). Activation of PPARA-mediated autophagy reduces Alzheimer disease-like pathology and cognitive decline in a murine model. Autophagy.

[18] Raha S, Ghosh A, Dutta D, Patel DR, Pahan K (2021). Activation of PPARα enhances astroglial uptake and degradation of β-amyloid. Sci Signal.

[19] Qu X-X, He J-H, Cui Z-Q, Yang T, Sun X-H (2022). PPAR-α agonist GW7647 protects against oxidative stress and Iron Deposit via GPx4 in a transgenic mouse model of Alzheimer’s Diseases. ACS Chem Neurosci.

[20] Oh E (2022). Synthetic PPAR agonist DTMB alleviates Alzheimer’s Disease pathology by inhibition of chronic microglial inflammation in 5xFAD mice. Neurotherapeutics.

[21] Chandra S, Pahan K (2019). Gemfibrozil, a lipid-lowering drug, lowers amyloid plaque pathology and enhances memory in a mouse model of Alzheimer’s disease via peroxisome proliferator-activated receptor α. J Alzheimers Dis Rep.

[22] Settembre C, Fraldi A, Medina DL, Ballabio A (2013). Signals from the lysosome: a control centre for cellular clearance and energy metabolism. Nat Rev Mol Cell Biol.

[23] Xiao Q (2014). Enhancing astrocytic lysosome biogenesis facilitates Abeta clearance and attenuates amyloid plaque pathogenesis. J Neurosci.

[24] Polito VA (2014). Selective clearance of aberrant tau proteins and rescue of neurotoxicity by transcription factor EB. EMBO Mol Med.

[25] Xiao Q (2015). Neuronal-targeted TFEB accelerates lysosomal degradation of APP, reducing Abeta generation and amyloid plaque pathogenesis. J Neurosci.

[26] Martini-Stoica H (2018). TFEB enhances astroglial uptake of extracellular tau species and reduces tau spreading. J Exp Med.

[27] Xu Y (2021). TFEB regulates lysosomal exocytosis of tau and its loss of function exacerbates tau pathology and spreading. Mol Psychiatry.

[28] Astarita G (2006). Pharmacological characterization of hydrolysis-resistant analogs of oleoylethanolamide with potent anorexiant properties. J Pharmacol Exp Ther.

[29] Ghosh A (2020). An epoxide hydrolase inhibitor reduces neuroinflammation in a mouse model of Alzheimer’s disease. Sci Transl Med.

[30] Nezich CL, Wang C, Fogel AI, Youle RJ (2015). MiT/TFE transcription factors are activated during mitophagy downstream of parkin and Atg5. J Cell Biol.

[31] Lian H, Roy E, Zheng H (2016). Protocol for primary microglial culture preparation. Bio Protoc.

[32] Litvinchuk A (2018). Complement C3aR inactivation attenuates tau pathology and reverses an immune network deregulated in tauopathy models and Alzheimer’s disease. Neuron.

[33] Swartzlander DB (2018). Concurrent cell type-specific isolation and profiling of mouse brains in inflammation and Alzheimer’s disease. JCI Insight.

[34] Lian H, Roy E, Zheng H (2016). Microglial phagocytosis assay. Bio Protoc.

[35] Pascual G (2017). Targeting metastasis-initiating cells through the fatty acid receptor CD36. Nature.

[36] Gedam M (2023). Complement C3aR depletion reverses HIF-1α–induced metabolic impairment and enhances microglial response to Aβ pathology. J Clin Invest.

[37] Lee SS (1995). Targeted disruption of the alpha isoform of the peroxisome proliferator-activated receptor gene in mice results in abolishment of the pleiotropic effects of peroxisome proliferators. Mol Cell Biol.

[38] Settembre C (2011). TFEB links autophagy to lysosomal biogenesis. Sci (1979).

[39] Ghosh A (2015). Activation of peroxisome proliferator-activated receptor α induces lysosomal biogenesis in brain cells. J Biol Chem.

[40] Napolitano G (2018). mTOR-dependent phosphorylation controls TFEB nuclear export. Nat Commun.

[41] Settembre C (2013). TFEB controls cellular lipid metabolism through a starvation-induced autoregulatory loop. Nat Cell Biol.

[42] Laplante M, Sabatini DM (2012). mTOR Signaling in growth control and disease. Cell.

[43] Palmieri M (2017). mTORC1-independent TFEB activation via akt inhibition promotes cellular clearance in neurodegenerative storage diseases. Nat Commun.

[44] Guijarro A, Fu J, Astarita G, Piomelli D (2010). CD36 gene deletion decreases oleoylethanolamide levels in small intestine of free-feeding mice. Pharmacol Res.

[45] Gervois P (2004). Global suppression of IL-6-induced acute phase response gene expression after chronic in vivo treatment with the peroxisome proliferator-activated receptor-α activator fenofibrate. J Biol Chem.

[46] Bougarne N (2018). Molecular actions of PPARα in lipid metabolism and inflammation. Endocr Rev.

[47] Zhang X, Liu W, Zan J, Wu C, Tan W (2020). Untargeted lipidomics reveals progression of early Alzheimer’s disease in APP/PS1 transgenic mice. Sci Rep.

[48] Prakash P (2021). Monitoring phagocytic uptake of amyloid β into glial cell lysosomes in real time. Chem Sci.

[49] Marschallinger J (2020). Lipid-droplet-accumulating microglia represent a dysfunctional and proinflammatory state in the aging brain. Nat Neurosci.

[50] Oakley H (2006). Intraneuronal beta-amyloid aggregates, neurodegeneration, and neuron loss in transgenic mice with five familial Alzheimer’s disease mutations: potential factors in amyloid plaque formation. J Neurosci.

[51] Xiao N-A (2015). Reduction of glucose metabolism in olfactory bulb is an earlier Alzheimer’s Disease-related Biomarker in 5XFAD mice. Chin Med J (Engl).

[52] Andersen JV (2021). Hippocampal disruptions of synaptic and astrocyte metabolism are primary events of early amyloid pathology in the 5xFAD mouse model of Alzheimer’s disease. Cell Death Dis.

[53] Liddelow SA (2017). Neurotoxic reactive astrocytes are induced by activated microglia. Nature.

[54] Claes C (2021). Plaque-associated human microglia accumulate lipid droplets in a chimeric model of Alzheimer’s disease. Mol Neurodegener.

[55] de la Monte SM, Wands JR (2006). Molecular indices of oxidative stress and mitochondrial dysfunction occur early and often progress with severity of Alzheimer’s disease. J Alzheimer’s Disease.

[56] Sáez-Orellana F (2021). Regulation of PPARα by APP in Alzheimer disease affects the pharmacological modulation of synaptic activity. JCI Insight.

[57] Overton HA (2006). Deorphanization of a G protein-coupled receptor for oleoylethanolamide and its use in the discovery of small-molecule hypophagic agents. Cell Metab.

[58] Tellez LA (2013). A gut lipid messenger links excess dietary fat to dopamine deficiency. Sci (1979).

[59] Roy A (2016). Identification and characterization of PPARα ligands in the hippocampus. Nat Chem Biol.

[60] Kim S-M (2017). TREM2 promotes Aβ phagocytosis by upregulating C/EBPα-dependent CD36 expression in microglia. Sci Rep.

