# Supplementary material for: Oleoylethanolamide facilitates PPARα and TFEB signaling and attenuates Aβ pathology in a mouse model of Alzheimer’s disease
Source: Mol Neurodegener. 2023 Aug 15;18:56. doi: 10.1186/s13024-023-00648-x (PMC10426131; doi:10.1186/s13024-023-00648-x)
Supplement: Supplementary file 1 — Supplementary Material 1 [file 13024_2023_648_MOESM1_ESM.docx]

**Supplemental Materials for**

**Oleoylethanolamide facilitates PPARα and TFEB signaling and attenuates Aβ pathology in a mouse model of Alzheimer’s disease**

Michele M. Comerota^1^, Manasee Gedam^1,2^, Wen Xiong^1^, Feng Jin^1,3^, Lisheng Deng^3^, Meng C. Wang^1,4,5*^, Jin Wang^3^ and Hui Zheng^1,2,4**^

^1^Huffington Center on Aging, ^2^Translational Biology and Molecular Medicine Graduate Program, ^3^Department of Pharmacology and Chemical Biology, ^4^Department of Molecular and Human Genetics, ^5^Howard Hughes Medical Institute, Baylor College of Medicine, Houston TX, USA

- **Supplementary Methods**
- **Supplementary Figures and Legends**

Figure S1. Changes of PPARα pathway in aging and AD.

Figure S2. Characterization of PPARα and TFEB pathways in mouse brains and sorted microglia.

Figure S3. Effect of KDS-5104 on microglial gene expression and Akt signaling.

Figure S4. Phagocytosis genes were upregulated by KDS-5104 treatment.

Figure S5: Efficacy and safety studies of KDS-5104 dosing.

Figure S6. KDS-5104 increases CD36 and suppresses lipid and DAM signatures.

Figure S7. KDS-5104 treatment does not alter ADAM10 and APP levels.

Figure S8. General neurological assessment of KDS-5104 treatment.

Figure S9. No sex dependent differences in KDS-5104’s effect on amyloid pathology or behavior in 5xFAD mice.

- **Supplementary Tables**

Table S1. Demographic data for postmortem brain tissue from AD and aged matched controls.

Table S2. Significantly upregulated lipid species by KDS-5104 treatment in 5xFAD mice.

Table S3. List of primers for qPCR.

Table S4. List of antibodies.

**Supplementary Methods**

***Human brain specimens***

Postmortem brain tissues from AD patients and non-demented controls were provided by the University of Pennsylvania Center for Neurodegenerative Disease Research (CNDR). Informed consent was obtained from all subjects for the use of their postmortem tissues. The demographic data for the human AD and control can be found in Supplementary Table 1.

***Subchronic KDS-5104 treatment***

For in vivo KDS-5104 dosage determination and safety studies we used 2-month-old WT mice treated every other day for 3 weeks with two doses of KDS-5104 (10 mg/kg and 50 mg/kg, i.p.). Vehicle was 0.1% ethanol in PBS. Mice were examined for overall activities before and after each injection. At the conclusion of the treatment, general neurological assessments were performed, and mice were sacrificed. Brains were quickly extracted, fixing half in 4% paraformaldehyde (PFA) for immunofluorescent staining and flash freezing the other half for molecular and biochemical analyses. Liver tissue was also collected for analysis of peripheral changes. ***Behavioral analysis***

*Open field Arena:* Each mouse was placed singly in the center of the open field arena apparatus (OmniTech Electronics, Columbus, OH). Mice were allowed to freely move around the apparatus for 30 min while locomotion activity was recorded using the Versamax activity monitoring software (AccuScan Instruments, Columbus, OH).

*Grip Strength:* Each mouse was held by the tail near the grid bar of a digital grip strength meter (Columbus Instruments, Columbus, OH) and allowed to fully clasp the grid with both forepaws. The mouse was then pulled parallel away from the meter with a constant force until both forepaws released. The grip strength, measured in kg of force was recorded. The procedure was performed for a total of three trials, averaging the three forces for the final result per mouse.

*Rotarod:* Coordination and motor function were measured by using an accelerated rotating rod test (type 7650; Ugo Basile, Milan, Italy). Mice were placed on the rod (3 cm diameter, 30 cm long) for four trials, with each trial lasting 5 minutes. The rod accelerated from 4 to 40 rpm in 5 min. Latency to fall for each mouse was recorded.

**Supplementary Figures and Legends**

**
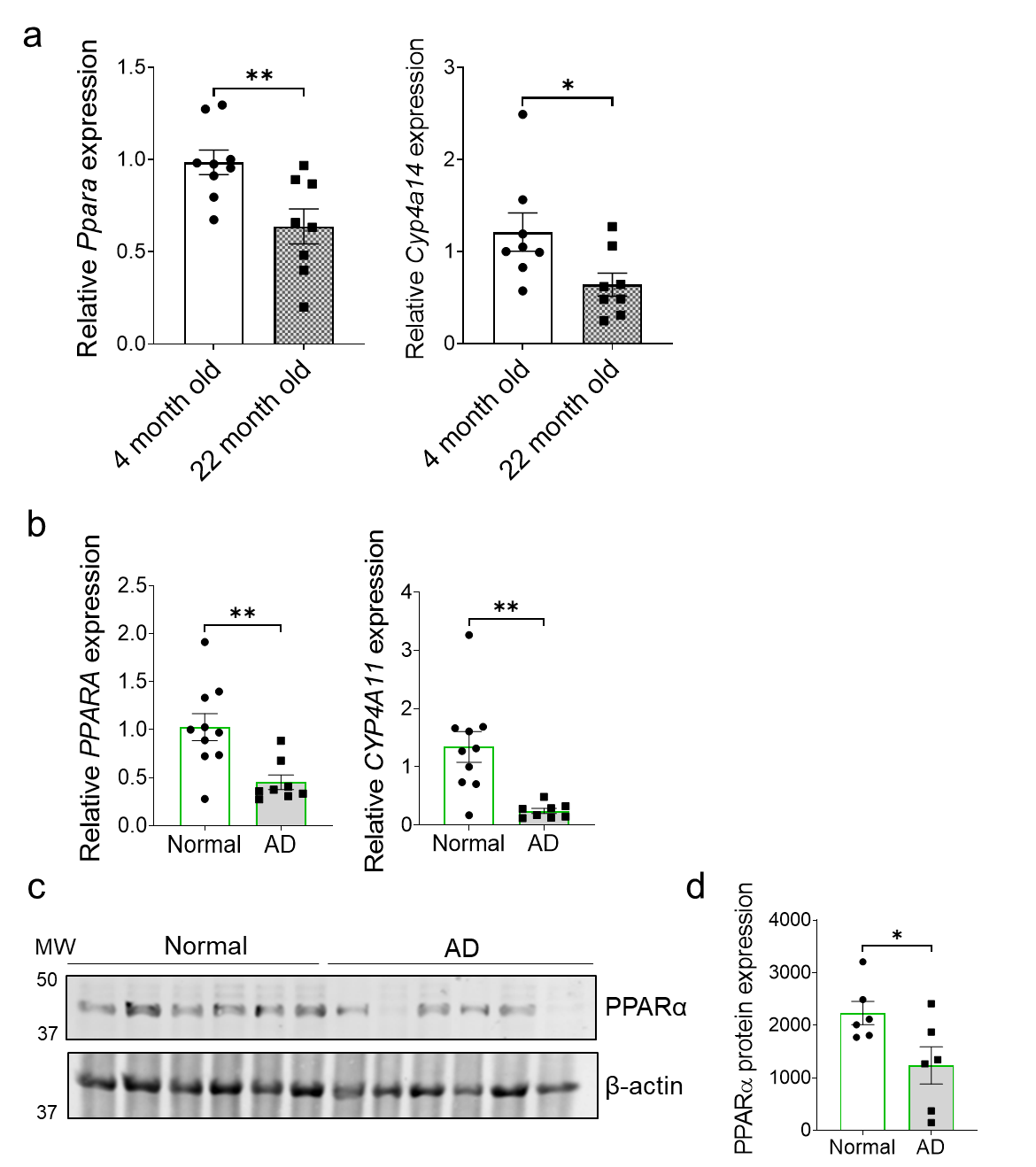
**

**Figure S1. Changes of PPARα pathway in aging and AD.**

**a.** qPCR analysis of *Ppara* and downstream target *Cyp4a14* in the cortex of 4- and 22-month-old mice *(n=8/group)*. **b.** qPCR analysis of expression of *PPARA* and downstream target *CYP4A11* in the cortex of human AD patients and aged matched controls *(n=8-10/group)*. **c.** Western blot analysis of the expression of PPARα in postmortem brain tissue from AD patients and cognitively normal aged-matched controls (*n=6/group*). β-actin was used as a loading control. **d.** Quantification of PPARα protein levels normalized to β-actin. For all panels, data are presented as mean ± SEM. **p* < 0.05, ***p* < 0.01 by 2-sided *t* tests.

**
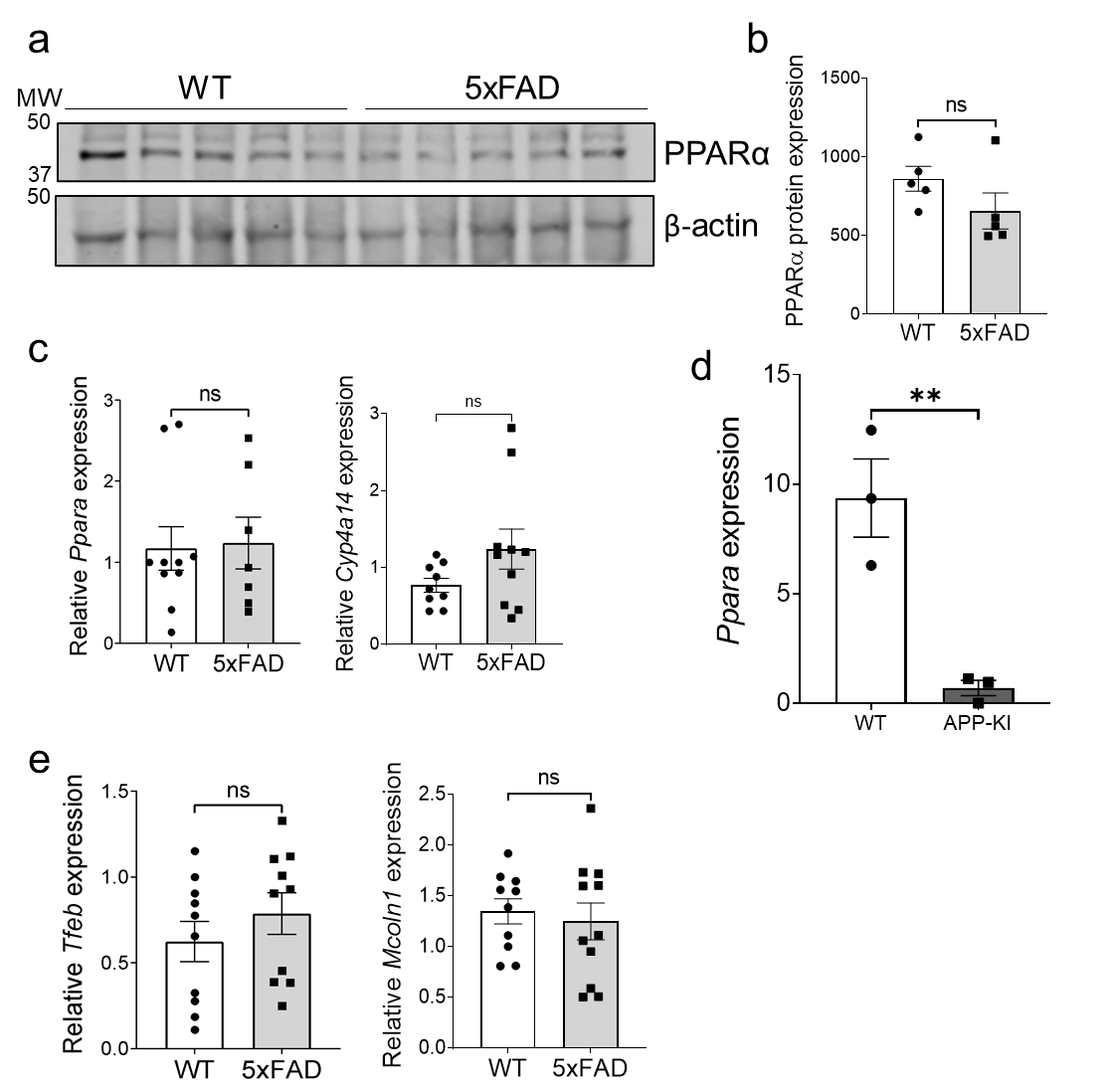
**

**Figure S2. Characterization of PPARα and TFEB pathways in mouse brains and sorted microglia.**

**a.** Representative Western blot analysis of PPARα in 9-month-old 5xFAD and WT littermates *(n=5/group)*. Loading control was β-actin. **b.** Quantification of PPARα protein levels normalized to β-actin. **c.** qPCR analysis of expression of *Ppara* and downstream target *Cyp4a14* in the cortex of 9-month-old 5xFAD and WT littermates *(n=10/group)*. **d.** *Ppara* expression from microglia sorted from 9-month-old APP-KI mice compared to WT *(n=3/group)*. **e.** qPCR analysis of *Tfeb* and *Mcoln1* in the cortex of 9-month-old 5xFAD and WT littermates *(n=10/group)*. For all panels, data are presented as mean ± SEM. ns: non-significant, ***p* < 0.01, by 2-sided *t* tests.


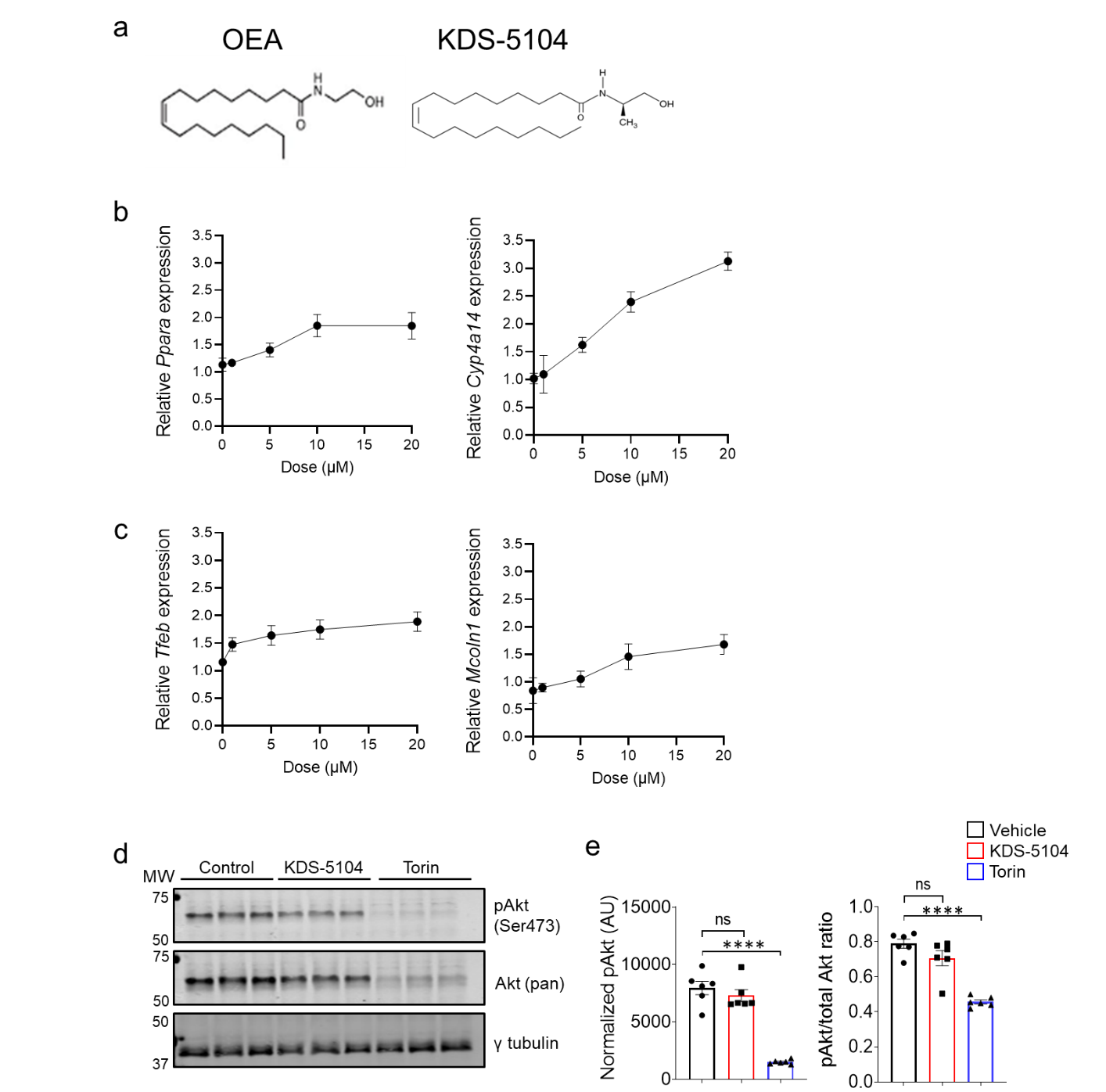


**Figure S3. Effect of KDS-5104 on microglial gene expression and Akt signaling.**

**a.** Structure depiction of OEA and KDS-5104. **b.** qPCR analysis of *Ppara* and *Cyp4a14* in primary microglia treated with various concentrations of KDS-5104 (0 µM, 1 µM, 5 µM, 10 µM, and 20 µM) (*n=3/condition*). **c.** qPCR analysis of *Tfeb* and *Mcoln1* in microglia treated with various concentrations of KDS-5104 (0 µM, 1 µM, 5 µM, 10 µM, and 20 µM) (*n=3/condition*). **d.** Representative Western blot analysis of pAkt (Ser473 phosphorylation site), total Akt in KDS-5104 treated microglia. γ tubulin was used as a loading control. **e.** Quantification of pAkt normalized to tubulin and ratio of pAkt to total Akt (*n=5/group*). Data are presented as mean ± SEM. ns: non-significant, *****p* < 0.0001. One way ANOVA with Tukey's multiple comparisons tests as the post hoc analysis.


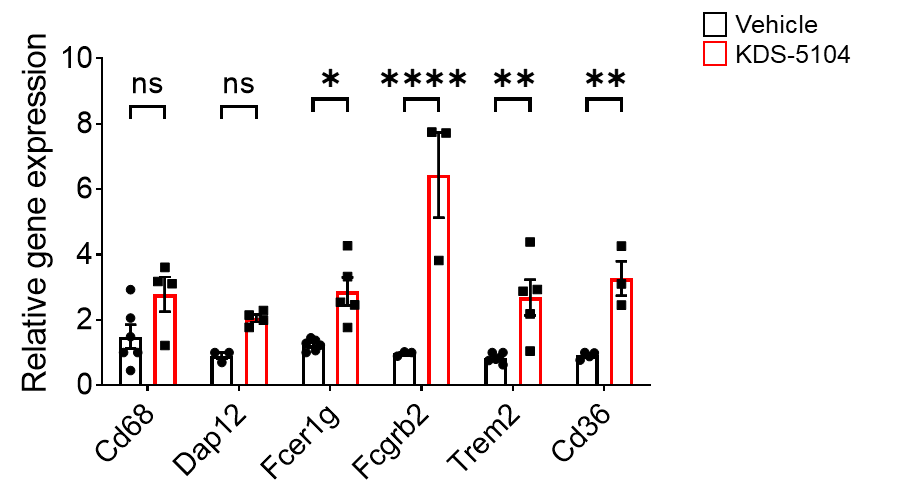


**Figure S4. Phagocytosis genes were upregulated by KDS-5104 treatment.**

qPCR analysis of phagocytosis genes in WT primary microglia treated with KDS-5104 (10 µM, 8 hours) compared to vehicle treated control, showing *Cd68* and *Dap12* trending upwards but not significant while *Fcer1g*, *Fcgr2b*, *Trem2* and *Cd36* are significantly upregulated (*n=4-5/group*). Data are presented as mean ± SEM. ns: non-significant, **p*< 0.05, *** p*<0.01*,* **** *p*<0.001 by two-sided *t*-tests.


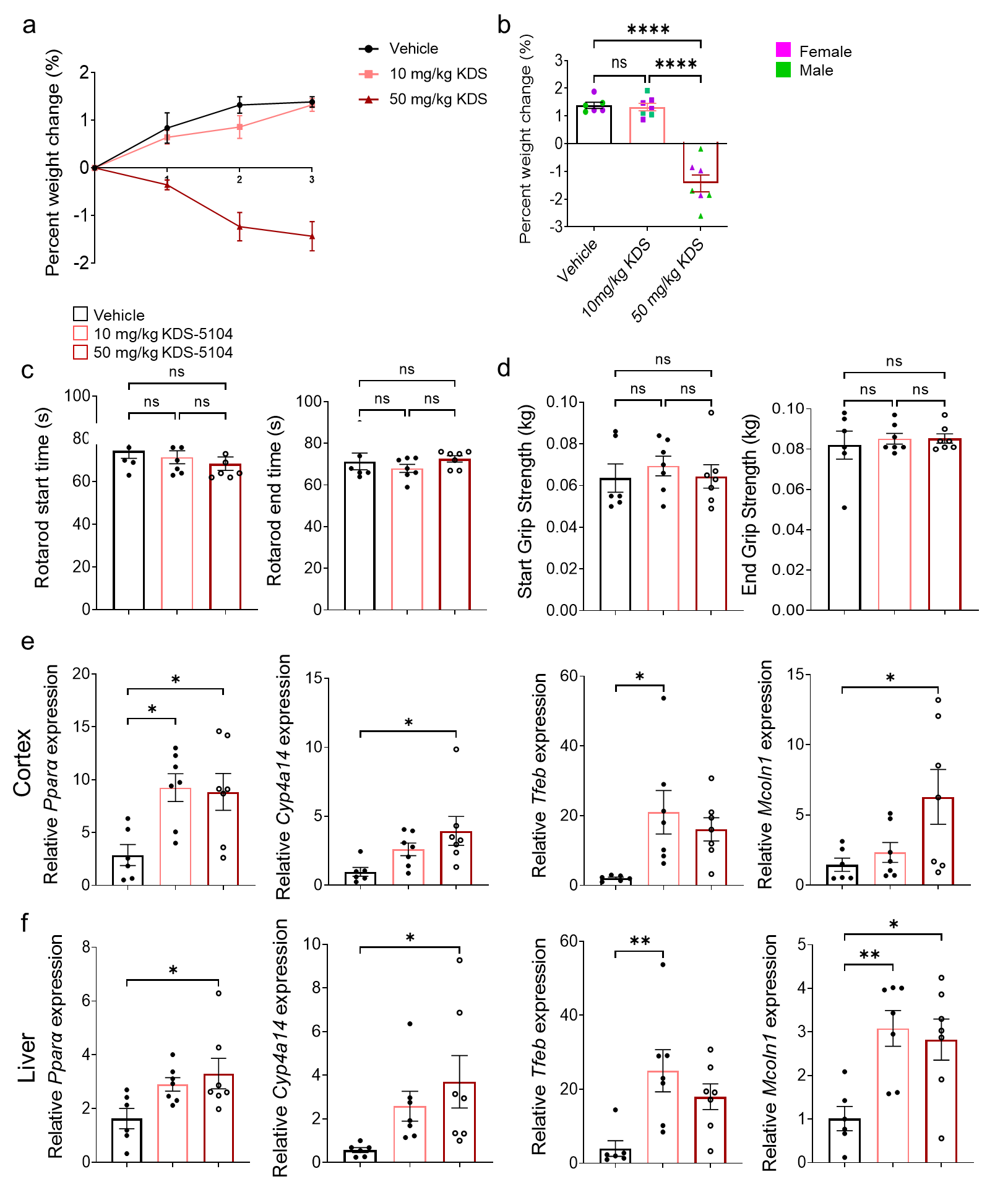


**Figure S5. Efficacy and safety studies of KDS-5104 dosing.**

**a.** Measurement of body weight changes of WT mice i.p. injected with vehicle, 10 mg/kg or 50 mg/kg KDS-5104 once of every other day for three weeks. **b.** Final body weight changes separated by male and female. **c** and **d**. Rotarod (c) and grip strength (d) tested prior to and at the end of treatment. **e.** qPCR analysis of *Ppara*, *Cyp4a14, Tfeb* and *Mcoln1* in cortical samples of WT mice with vehicle, 10 mg/kg or 50 mg/kg KDS-5104 treatment. **f.** Same as (e) except liver tissue was analyzed. n=5-7 mice/group was used for each experiment. Data are presented as mean ± SEM. ns: non-significant, **p* < 0.05, ***p* < 0.01, *****p* < 0.0001. One way ANOVA with Tukey's multiple comparisons tests as the post hoc analysis.


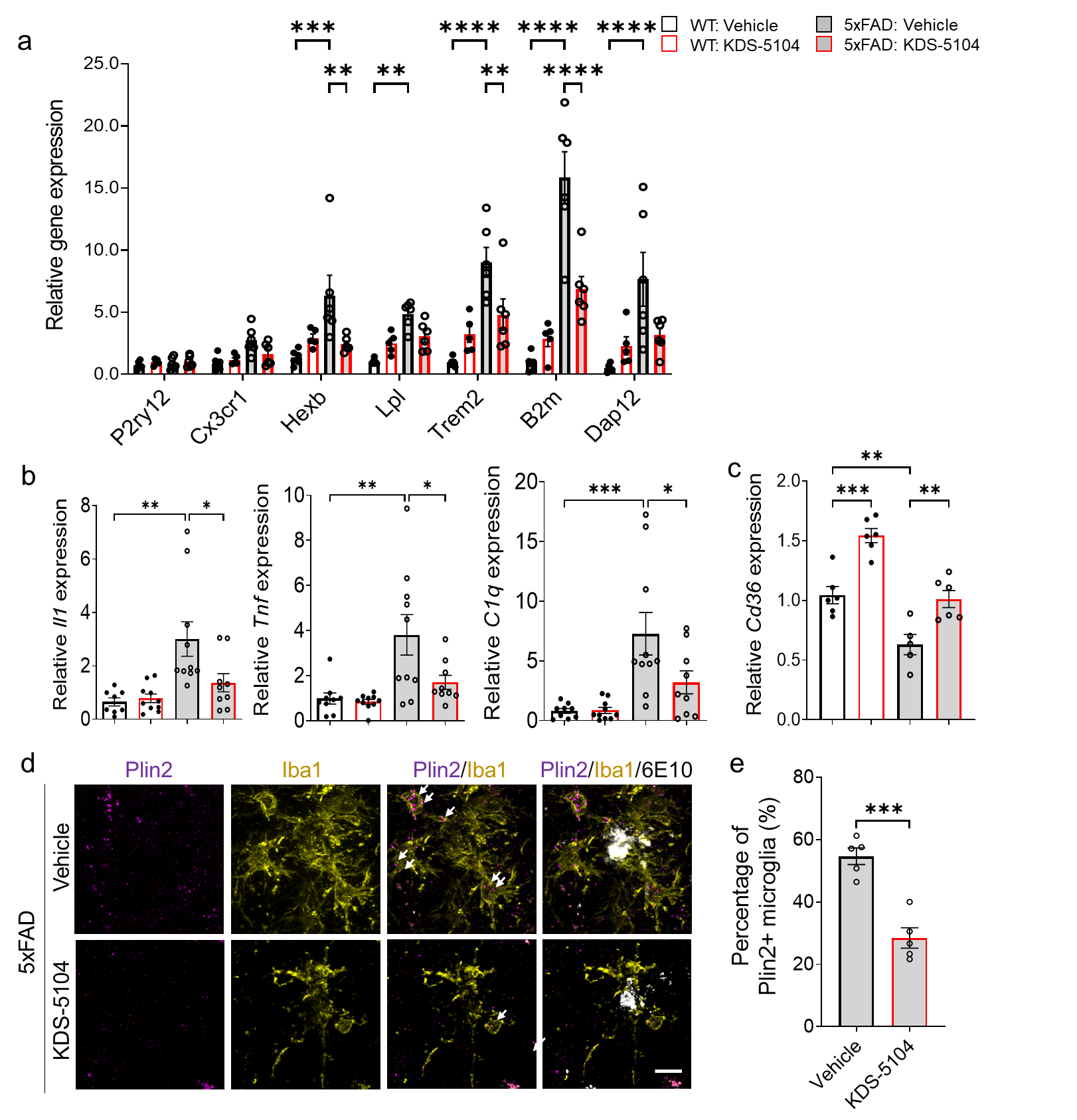


**Figure S6. KDS-5104 suppresses lipid and DAM signatures.**

**a.** qPCR analysis of homeostatic (*P2ry12* and *Cx3cr1*) and disease associated microglia markers (*Hexb, Lpl,* *Trem2, B2m* and *Dap12)* in 4-month-old WT and 5xFAD mice treated with vehicle or 10 mg/kg KDS-5104 for 2 months *(n=5-6/group).* **b.** qPCR analysis of *Il1*, *Tnf* and *C1qa* expression in 4-month-old WT and 5xFAD treated with vehicle or KDS-5104 *(n=9 mice/group)*. **c.** qPCR analysis of *Cd36* expression in 4-month-old WT and 5xFAD treated with vehicle or KDS-5104 *(n=5-6 mice/group)*. **d.** Representative images of Plin2 (magenta), Iba1 (yellow) and 6E10 (white) co-staining from hippocampal sections of 4-month-old 5xFAD mice treated with vehicle or KDS-5104. Scale bar: 10 μm. **e.** Quantification of the percentage of microglia that are Plin2 positive *(n=5 mice/group).* Data are presented as mean ± SEM. **p* < 0.05, ***p* < 0.01, ****p* < 0.001, *****p* < 0.0001 by 2-sided *t* test (d) or by one way ANOVA with Tukey's multiple comparisons tests as the post hoc analysis (a, b, e).

**Figure S7. KDS-5104 treatment does not alter ADAM10 and APP levels.**

**a.** Western blot analysis of ADAM10 and APP full length (APP-FL) and C-terminal fragments (APP-CTF) in the cortical protein extract from vehicle or KDS-5104 treated 5xFAD mice. β-actin was used as a loading control. Quantification of **(b)** ADAM 10 precursor and active form, **(c)** APP-FL and APP-CTF normalized to β-actin *(n=6 mice/group)*. Data are presented as mean ± SEM. ns: non-significant by 2-sided *t* tests.

**
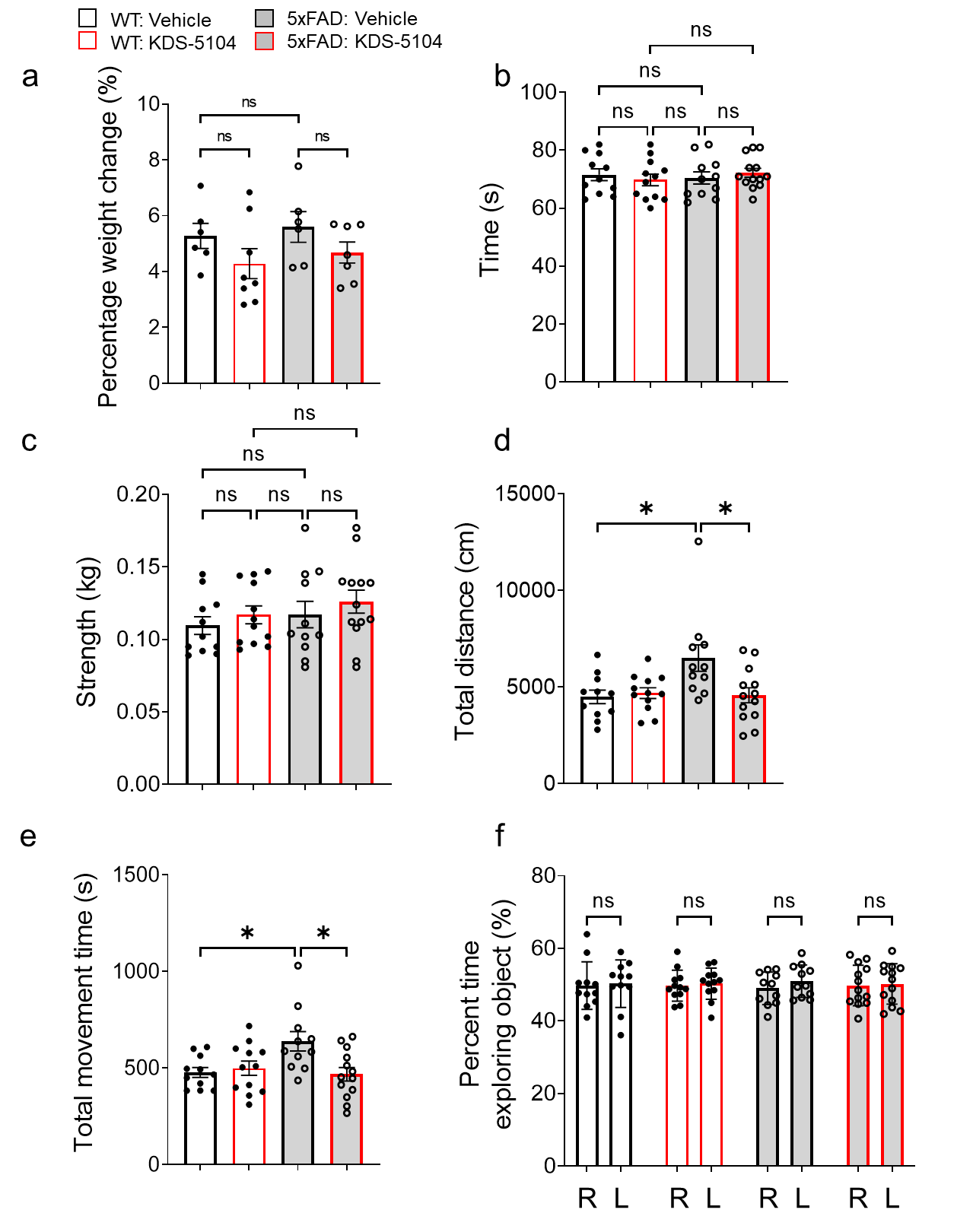
**

**Figure S8. General neurological assessment of KDS-5104 treatment.**

**a.** Bar graph depicting the total weight change of WT and 5xFAD mice treated with vehicle or KDS-5104 (10 mg/kg) for 2 months *(n=6-8 mice/group)*. **b**. Bar graph depicting the time spent on rotarod in WT and 5xFAD mice treated with vehicle or KDS-5104. **c.** Bar graph showing the fore limb force (kg) of each animal measured by grip strength apparatus. The open field test to assess total distance travelled (**d**) and total movement time (**e**). **f.** Bar graph depicting the percentage of time spent with each identical object in the training phase of NOR for each group *(n=8-10 mice/group)*. Data are presented as mean ± SEM. ns: non-significant. **p* < 0.05. One way ANOVA with Tukey's multiple comparisons tests as the post hoc analysis.

**
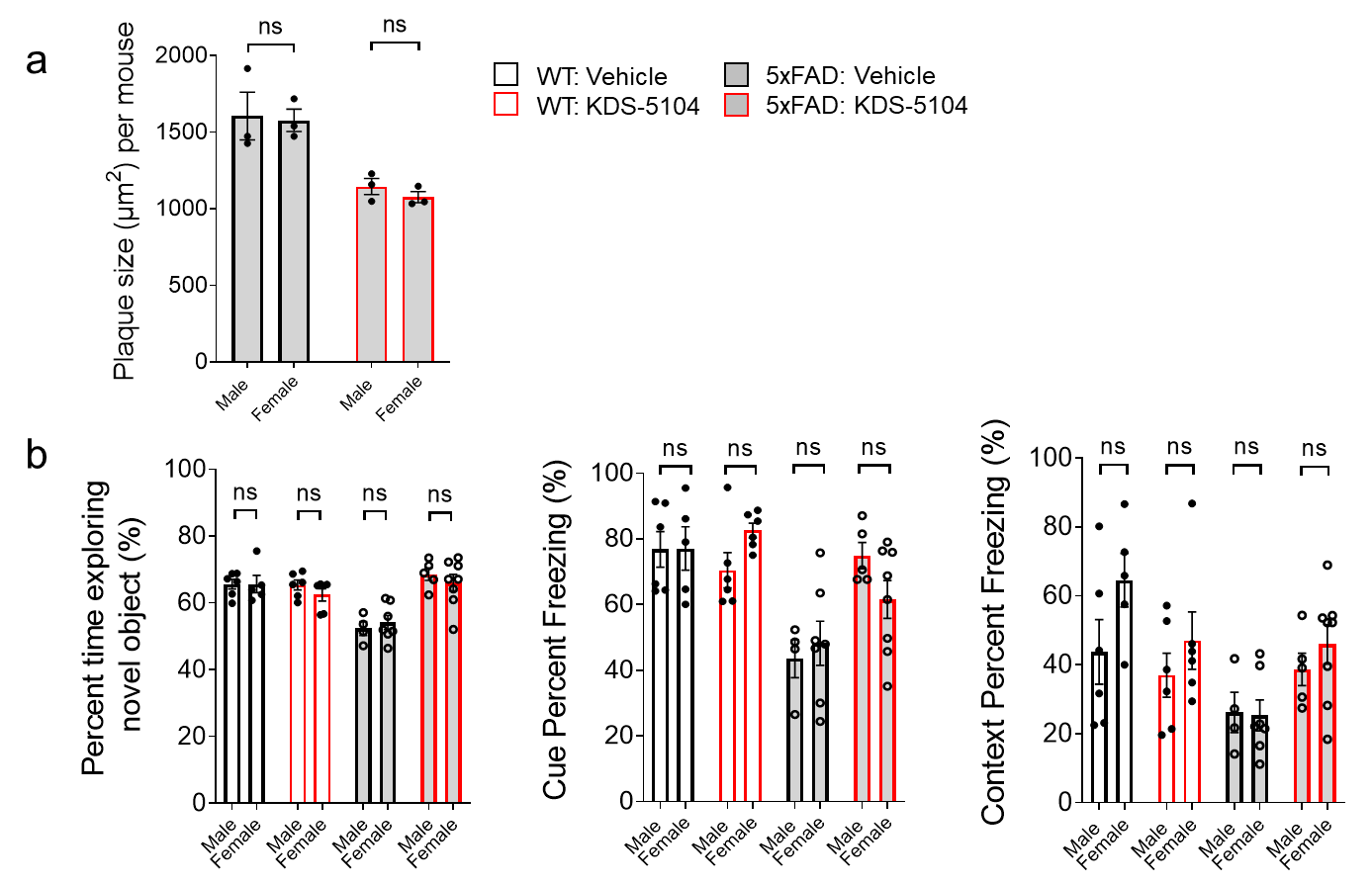
**

**Figure S9. No sex dependent differences in KDS-5104’s effect on amyloid pathology or behavior in 5xFAD mice.**

Two-way ANOVA test with sex as a between-subjects factor and treatment group as a within-subjects factor showed no significant differences in sex or sex x treatment interaction in (**a**) Aβ pathology (*n=3/sex*) and (**b**) behavior (novel object recognition and fear conditioning) (*n=6-8/sex*). Data are presented as mean ± SEM. ns: non-significant.

**Table S1. Demographic data for postmortem brain tissue from AD patients and aged matched cognitively normal controls.**

| **INDDID** | **Diagnosis** | **Sex** | **Age** |
| --- | --- | --- | --- |
| 117504 | Normal | Male | 59 |
| 113695 | Normal | Female | 59 |
| 102215 | Normal | Female | 65 |
| 100786 | Normal | Male | 61 |
| 101799 | Normal | Male | 70 |
| 103376 | Normal | Female | 68 |
| 107712 | Normal | Male | 70 |
| 110602 | Normal | Male | 55 |
| 113818 | Normal | Female | 65 |
| 116519 | Normal | Female | 67 |
| 109650 | AD | Female | 64 |
| 115165 | AD | Male | 64 |
| 120851 | AD | Male | 64 |
| 106029 | AD | Male | 71 |
| 121576 | AD | Male | 69 |
| 101034 | AD | Male | 67 |
| 116620 | AD | Female | 55 |
| 112723 | AD | Male | 72 |

**Table S2. Statistically significantly upregulated lipid species by KDS-5104 treatment in 5xFAD mice.**


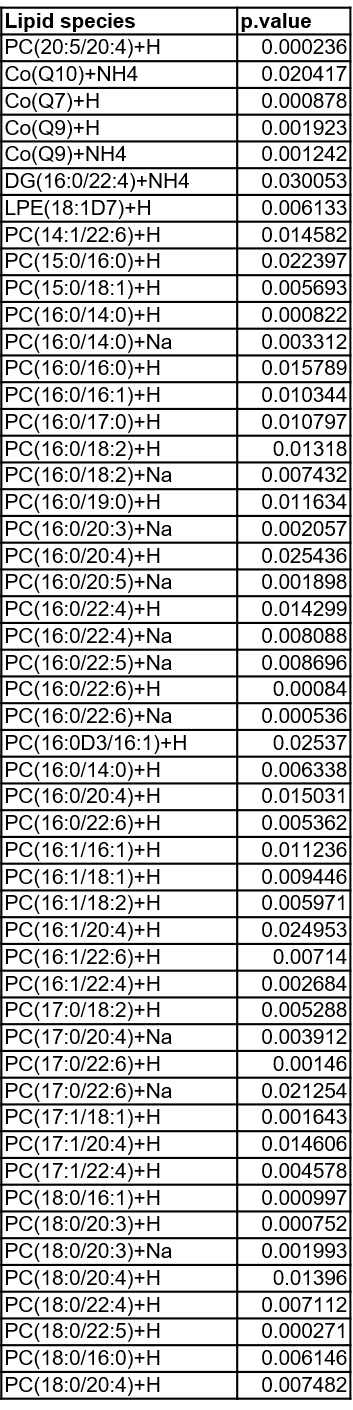

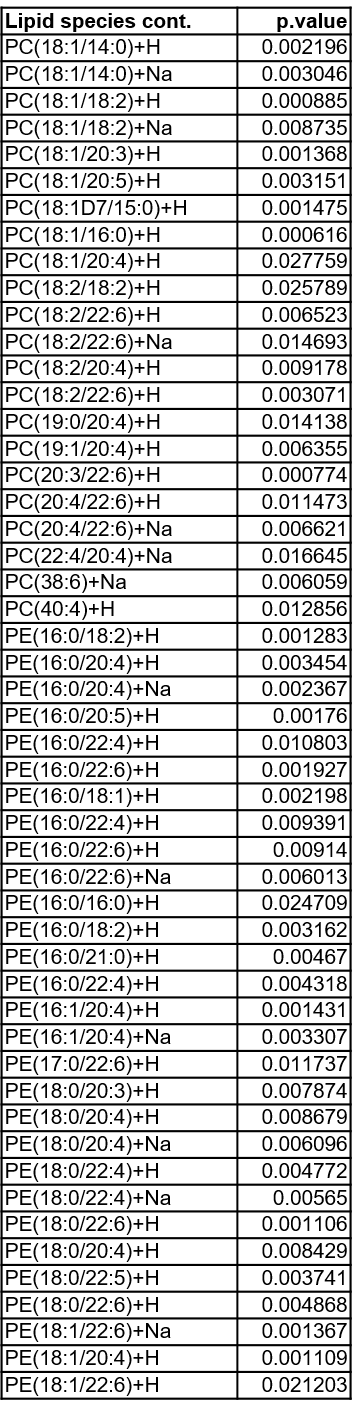

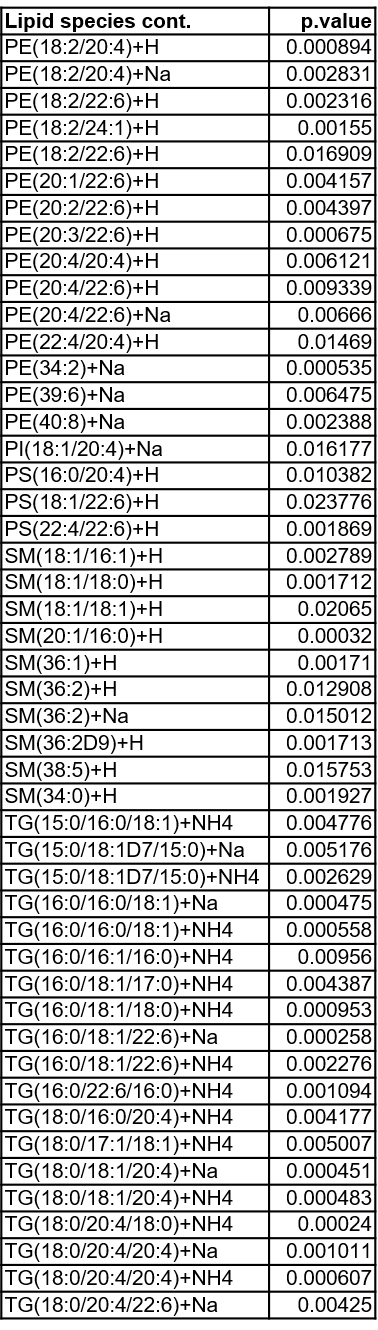


**Table S3. List of primer sequences for qPCR.**
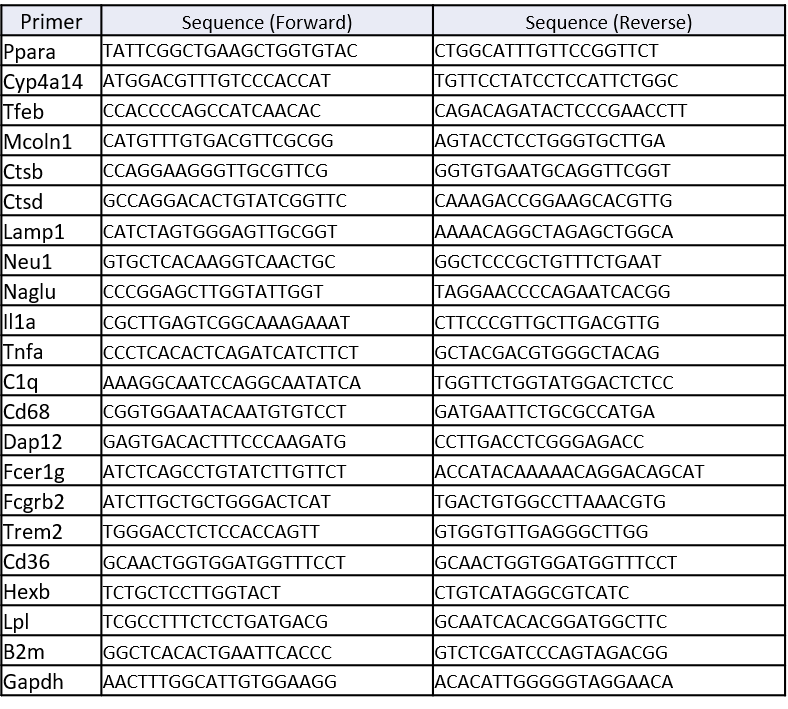

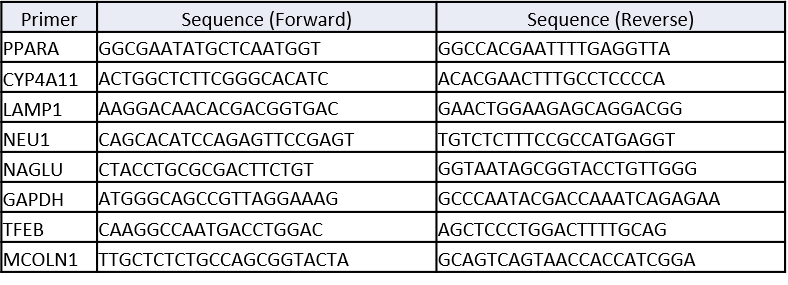


Mouse

Human

**Table S4. List of antibodies.**

| Antibody | Source | Catalog number |
| --- | --- | --- |
| Rabbit ant-pS6K (clone: 108D2) | Cell Signaling | 9234S |
| Rabbit anti-S6K | Cell Signaling | 9202S |
| Mouse anti-. tubulin (clone: GTU-88) | Sigma | T6557 |
| Rat anti-Lamp1 (clone: 1D4B) | BD Biosciences | 553792 |
| Mouse anti-CD36 (clone: JC63.1) | Abcam | 23680 |
| Goat anti-Iba1 | Novus Biologicals | NB100-1028 |
| Rabbit anti-Iba1 | Wako | 019-19741 |
| Mouse anti-GFAP (clone: GA5; Millipore; Cat#MAB360) | Millipore | MAB360 |
| Rabbit anti-ASC (clone: AL177) | Adipogen | AG-25B-006-C100 |
| Mouse anti-Aß (clone: 6E10) | Biolegend | 803001 |
| Rat anti-CD68 (clone: FA-11) | BioRad | MCA1957GA |
| Mouse anti-PSD95 (clone: 6G6-1C9) | Millipore | MAB1596 |
| Rabbit anti-synaptophysin | Abcam | ab16659 |
| Rabbit anti-PPARa | Invitrogen | PA1-822A |
| Mouse anti-beta actin (clone: AC-74) | Sigma | A5316 |
| Rabbit anti-pAKT (clone: S473) | Cell Signaling | 9271S |
| Rabbit ant-AKT | Cell Signaling | 4691S |
| Rabbit anti-APP | Cell Signaling | 2452 |
| Rat anti mouse CD11b-FITC | BD Biosciences | 563890 |
| Rat anti mouse CD11b-FITC | BD Biosciences | 553310 |
| Rabbit anti Perilipin 2 | Novus | NB110-40877SS |
| Rabbit anti ADAM10 | Cell Signaling | 14194 |
